# In defence of ferroptosis

**DOI:** 10.1038/s41392-024-02088-5

**Published:** 2025-01-03

**Authors:** Francesca Alves, Darius Lane, Triet Phu Minh Nguyen, Ashley I. Bush, Scott Ayton

**Affiliations:** 1 The Florey Institute of Neuroscience and Mental Health, Melbourne, VIC Australia; 2 Florey Department of Neuroscience and Mental Health, The University of Melbourne, Melbourne, VIC Australia

**Keywords:** Cell death in the nervous system, Cell biology, Neurological disorders

## Abstract

Rampant phospholipid peroxidation initiated by iron causes ferroptosis unless this is restrained by cellular defences. Ferroptosis is increasingly implicated in a host of diseases, and unlike other cell death programs the physiological initiation of ferroptosis is conceived to occur not by an endogenous executioner, but by the withdrawal of cellular guardians that otherwise constantly oppose ferroptosis induction. Here, we profile key ferroptotic defence strategies including iron regulation, phospholipid modulation and enzymes and metabolite systems: glutathione reductase (GR), Ferroptosis suppressor protein 1 (FSP1), NAD(P)H Quinone Dehydrogenase 1 (NQO1), Dihydrofolate reductase (DHFR), retinal reductases and retinal dehydrogenases (RDH) and thioredoxin reductases (TR). A common thread uniting all key enzymes and metabolites that combat lipid peroxidation during ferroptosis is a dependence on a key cellular reductant, nicotinamide adenine dinucleotide phosphate (NADPH). We will outline how cells control central carbon metabolism to produce NADPH and necessary precursors to defend against ferroptosis. Subsequently we will discuss evidence for ferroptosis and NADPH dysregulation in different disease contexts including glucose-6-phosphate dehydrogenase deficiency, cancer and neurodegeneration. Finally, we discuss several anti-ferroptosis therapeutic strategies spanning the use of radical trapping agents, iron modulation and glutathione dependent redox support and highlight the current landscape of clinical trials focusing on ferroptosis.

## Introduction

Ferroptosis is regarded as a cell death modality of metabolism. The biochemical mechanisms of ferroptosis involve a complex interaction between oxidative stress, lipid metabolism, and iron homeostasis that results in the peroxidation of polyunsaturated fatty acid (PUFA)-containing phospholipids to produce phospholipid peroxide radicals.^1–3^ These lipid peroxide radicals can react with other PUFAs that, in turn, generate additional peroxide radicals, which propagate as a chain-reaction throughout the phospholipid bilayer, leading to cell rupture. Briefly, ferroptosis-prone PUFAs contain bis-allylic hydrogen atoms that are liable to removal from the PUFA scaffold, exposing carbon moieties that can react with ferric iron or oxygen radicals either directly, or subsequent to attachment of molecular oxygen, form lipid (hydro)peroxides.^4^ The oxidation of PUFAs in the cell membrane can be initiated by reactive oxygen species (ROS) such as hydroxyl radicals, which can be generated by labile iron in the Fenton reaction.^5^ Hence, iron levels are strictly regulated by a variety of storage, transport and export proteins, including ferritin, transferrin, hepcidin, ferroportin and transferrin receptor 1 (TFR1) to avoid excess iron-derived ROS generation.^6^ Yet, the peroxidation of PUFAs is unavoidable, and this would result in ferroptosis if not continually interdicted by cellular defences. Thus, ferroptosis is distinct from other cell death modalities, where the initiation of cell death is an active event (e.g., apoptosis); ferroptosis, rather, usually is initiated by the withdrawal of cellular antioxidant defences.

Several antioxidant systems target upstream and downstream pathways of lipid peroxidation, with glutathione peroxidase 4 (GPX4) being the principal enzyme responsible for inhibiting ferroptosis.^7^ GPX4 is the only known enzyme that can detoxify phospholipid hydroperoxides directly in membranes.^8^ GPX4 consumes glutathione (GSH) when it performs this function, and it is supported by certain metabolites (e.g. ubiquinone) and vitamins (vitamins E, K, A) that can reduce phospholipid peroxides. These metabolites are recycled by enzymes that all consume nicotinamide adenine dinucleotide phosphate (NADPH): thioredoxin reductases (TR), glutathione reductase (GR), Ferroptosis suppressor protein 1 (FSP1), Dihydrofolate reductase (DHFR), NAD(P)H Quinone Dehydrogenase 1 (NQO1) and retinal reductases. Thus, NADPH is the foundational metabolite fuelling ant-ferroptotic defence through the above-mentioned metabolic intermediators. NADPH is depleted during ferroptosis and must be regenerated to avoid cell death.^1,9–12^ The reductive potential of NADPH is obtained by carbon metabolism, which underscores the inherent coupling of metabolism with ferroptosis.

After discussing the fundamentals of ferroptosis, including how it is modelled, where it occurs in the cell, and the role of lipids (the fuel of ferroptosis) and iron (the fire of ferroptosis), this review will focus on defence strategies to directly modulate and regulate lipids and iron in conjunction with key ferroptosis defence enzymes and metabolites that depend on NADPH. Subsequently, we will discuss evidence for ferroptosis in parallel with evidence for NADPH dysregulation in degenerative diseases.

## Modelling ferroptosis

In vitro models of ferroptosis have been developed to help understand the complex mechanisms underpinning this cell death modality. Key strategies to induce ferroptosis include 1.) system xc- inhibition by erastin or glutamate; which reduces cystine importation into cells leading to a lowering of intracellular glutathione. 2) Inhibition of glutathione synthesis by buthionine sulphoximine (BSO); which inhibits gamma-glutamylcysteine synthetase.^1,13,14^ 3.) GPX4 direct inhibition via RSL3, depletion by FIN56 or indirect inhibition by FINO2.^1,15–17^ 4.) iron-dependent lipid peroxidation; the addition of excess iron can induce ROS, lipid peroxidation and subsequent cell death, although toxicity is not always specific to ferroptosis^18,19^ and 5.) inhibition of ferroptosis suppressor protein 1 (FSP1) which prevents the production of reduced CoQ10 and other vitamins that possess lipophilic radical-trapping antioxidant properties.^20^

While existing models are useful tools to explore the biochemical mechanisms and risk factors leading to ferroptosis, there is a lack of translation into a clinical context, with no specific ferroptosis inhibitors or activators approved yet for clinical use. The measurement of ferroptosis in vivo is also limited by the lack a specific biomarker. Cell culture studies are often conducted in hyperoxic conditions with an abundance of metabolites exceeding physiological levels leading to a physiological translation gap. Cysteine deficiency is a foundational paradigm to initiate ferroptosis in vitro, yet we currently lack examples of chronic disease where cysteine deficiency is established. Apart from the brain, under physiological conditions system Xc-, composed of the transporter subunit, xCT (SLC7A11) and the regulatory subunit, SLC3A2, is modestly expressed in most tissues and *Slc7a11* knockout mice appear healthy with a normal lifespan and have no clear adverse phenotype.^21–24^ However, this changes when metabolism is hijacked in cancer with SCL7A11 levels rising to meet a new demand of cysteine for glutathione synthesis.^25^ Hence, new models of metabolic disturbances relevant to diseases are required to understand ferroptosis vulnerability and/or resistance which is likely underpinning manifestations of ferroptosis in vivo.

## Organelles involved in ferroptosis

Recent research has revealed that ferroptosis can be invoked and propagated by excessive lipid peroxidation at phospholipid-membrane-bound organelles, namely the mitochondria,^26^ endoplasmic reticulum,^27^ and Golgi apparatus.^28^ Since the peroxisome can synthesise and incorporate PUFA into the phospholipid membrane, peroxisomes may serve as not only a site of lipid peroxidation, but also a vehicle for propagating lipid peroxidation essential for ferroptosis progression.^29^ Conversely, increasing the number and size of lipid droplets (LD) is a strategy to sequester and shield PUFA from peroxidation, thus protecting the cells from ferroptosis.^30,31^ The activities of the nucleus, including (post)-transcriptional and cell cycle regulation, additionally serve to modulate ferroptosis in proliferative cancer cells. For instance, oxidative stress induces the translocation nuclear factor erythroid 2-related factor 2 (NRF2) from the cytosol to the nucleus where NRF2 transcriptionally activates the expression of enzymes involved in antioxidant defence system to minimise lipid peroxidation.^32,33^ It is also interesting to note that cell cycle arrest can either enhance ferroptosis sensitivity via stabilising p53 and CDK4/6 inhibition^34^ or promote resistance via inducing lipid droplet formation.^35^ As such, the role of nucleus in mediating either ferroptosis sensitivity or resistance appears context specific.

Like the nucleus, the lysosome plays a pleiotropic role in mediating ferroptosis. One of the first links between the lysosome and ferroptosis was drawn from the lysosomal-dependent autophagic process, which is regarded as an accelerator of ferroptosis via NCOA4-depedent ferritinophagy^36,37^ or RAB7A-dependent lipophagy, leading to the accumulation of reactive Fe^2+^ and lipid peroxides (see^38^ for a comprehensive review on autophagy-driven ferroptosis). Nonetheless, recent studies uncover that under cyst(e)ine deprivation, cells not only activate ATF4 stress response pathway to mobilise lysosomal cysteine storage,^39^ but also increase the uptake and breakdown of cysteine-rich albumin in the lysosome by cathepsin B (CTSB) to export of cysteine.^40^ Cysteine is then used as a substrate for the synthesis of GSH, which is essential for most antioxidant enzymatic activity. Thus, the lysosome plays an important role in maintaining the intracellular cysteine pool and so also serves as a checkpoint for ferroptosis. Together, these studies demonstrate that whether lysosome represents an accelerator or a brake for ferroptosis is context-specific, dependent on the types of stress.

Regardless of internal sites of lipid peroxidation, the termination of ferroptosis converges on the plasma membrane where cell rupture is facilitated by plasma membrane pores.^41,42^ Studies have also shown intercellular propagation of death following treatment with ferroptosis inducing agents.^1,11,43^ The concept of ferroptotic cell death propagation was recently explored in a muscle remodelling limb development system.^44^ Co et al. demonstrated that a ferroptotic death signal primed cells to become redox bistable enabling ROS amplification-diffusion events causing ferroptosis to spread via *trigger waves* (self-regenerating chemical fronts that spread rapidly over extended distances^45,46^) subsequently causing mass cell death. This study highlighted the critical need for ferroptosis defence systems to prevent tissue damage. Collectively, ferroptosis occurs because of a collapse of cellular antioxidant defence system leading to excessive lipid peroxidation at the phospholipid membrane of various internal organelles and plasma membrane.

## Lipid peroxidation and ferroptosis susceptibility

Ferroptosis occurs because of lipid peroxidation. The composition of phospholipids in the plasma membrane dictates ferroptosis vulnerability and the resources required to defend against it. These lipid classes are defined by double long-chain hydrocarbon attached to a glycerol backbone. The glycerol molecule contains a phosphate (3-position) that can be conjugated to different head groups. The four most common headgroups (choline, ethanolamine, serine and inositol) have different biophysical and chemical properties to provide diverse building blocks for a flexible asymmetric curved lipid bilayer: since ethanolamine is smaller than choline, ethanolamine head groups dominate in the inner leaflet of lipid bilayers, whereas the larger choline has a greater abundance in the outer leaflet.^47^ These favourable biophysical properties of phosphatidylethanolamines are complicated by an increased propensity toward lipid peroxidation. In addition to being located medially where they are exposed to intracellular free radicals and Fe^2+^, phosphatidylethanolamines often have a higher abundance of PUFA tail groups that are more susceptible to oxidation.^47^ Indeed, when ferroptosis is induced these are prominently oxidised.^48^

The biochemistry of lipid peroxidation was characterised over 25 years ago and consists of three key events 1.) Initiation 2.) Propagation cycles and 3.) Termination.^49–51^ Initiation occurs when an electron oxidant/free radical (i.e., hydroxyl, alkoxyl or hydroperoxyl radicals) abstracts a hydrogen atom from a lipid fatty acid to produce a carbon centred radical.^52^ Different phospholipid species have varying vulnerabilities to initiation due to variable degrees of difficulty in abstracting a hydrogen atom: polyunsaturated fatty acids (PL-PUFAs) are highly susceptible due to the presence of bis-allylic hydrogens that are more easily abstracted than hydrogens in monounsaturated fatty acids (MUFAs) or fully saturated fatty acids (SFAs). This carbon centred radical reacts with dioxygen to produce a lipid peroxyl radical L-OO⋅, or PL-PUFA-OO⋅ which is responsible for a series of propagation cycles since PL-PUFA-OO⋅ can produce new radicals by abstracting hydrogen from adjacent phospholipids forming PL-PUFA-OOH.^52^ PL-PUFA-OOHs are positioned at a ferroptosis-intersection whereby they can either 1.) react with labile Fe^2+^ (Fenton-like reaction) to produce a PL-PUFA-O⋅ which can propagate a peroxidation chain reaction on adjacent phospholipid, ultimately leading to ferroptosis or 2.) be converted by GPX4 to a lipid alcohol, a chemically inert species that is not susceptible to Fe^2+^ and radical propagation. Like the initiation step, the rate of propagation lipid peroxidation is influenced by the strength of carbon-hydrogen bond dissociation energies favouring weaker bonds. The weakest bonds are those at the bis-allylic methylene positions, followed by monoallylic hydrogen and alkyl C-H bonds.^49^ Substitution of hydrogen with deuterium atoms at the bis-allylic position reduces peroxidation susceptibility and consequently decreases ferroptosis vulnerability.^4^

Lipid peroxidation of membrane phospholipids is the executioner of ferroptosis. Using an oxidative lipidomics approach with florescent probes (BODIPY 581/591 and dihydrorhodamine 123), Vanden Berghe et al. (2020) distinguished ferroptosis from other regulated cell death modalities (apoptosis, necroptosis and pyroptosis) by demonstrating greater levels of lipid peroxidation and predominance of oxidised phosphatidylethanolamine species (oxPE) followed by oxidized phosphatidylserine (oxPS) and phosphatidylinositol (oxPI).^48^ Preferential oxidation of specific phospholipids in ferroptosis reveals that ferroptosis vulnerability depends on the presence or absence of oxidative-sensitive lipids. Hence, ferroptosis sensitivity is inextricably linked to phospholipid species composition and distribution.

### Modulation of phospholipid sensitivity to ferroptosis

Phospholipids can be synthesised de novo, but an efficient strategy to reduce (or increase) the ferroptosis risk of a phospholipid bilayer is via phospholipid remodelling. Through the Lands cycle, phospholipids can selectively substitute out and replace an acyl chain (sn-2 position). This occurs in two simplified stages 1.) phospholipases in the A2 family (PLA2) cleave the fatty acid at the sn-2 position to liberate a free fatty acid and lysophospholipid and 2.) a lysophospholipid acyl-transferase (LPLAT) esterifies the sn-2 position of the LPL with a new fatty acid.^53,54^ Acyl-CoA synthase long-chain (ACSL) family proteins are also required as they esterify CoA groups onto free fatty acids which enables their incorporation into phospholipids.

In 2015, Stockwell’s group conducted a genetic screen in haploid cells and discovered acyl-CoA synthetase long-chain family member 4 (ACSL4) and LPCAT3 as key proteins modulating lipid metabolism in ferroptosis.^55^ Similarly, MBOAT2 (also known as LPCAT4) was highlighted by a whole genome CRISPR activation screen as a ferroptosis supressing gene.^56^ ACSL4, LPCAT3 and MBOAT1/2 are enzymes that modulate polyunsaturated fatty acids (PUFAs) in membrane phospholipids, with varying phospholipid preferences, to modulate phospholipid sensitivity to ferroptosis.^12,55,57^ Specifically, MBOAT2 suppresses ferroptosis by selectively transferring MUFAs into Lyso-PE, thus decreasing availability of PE-PUFA, a preferred substrate for phospholipid peroxidation.^56^ LPCAT3 (also known as MBOAT5), preferentially introduces polyunsaturated acyl groups onto lyso-PC (sn-2 position) and ACSL4 (in conjunction with an LPCAT) sensitizes to ferroptosis by specifically esterifying arachidonic acid and adrenic acid into PE thus increasing the risk of oxidation and ferroptosis.^12,57,58^ Knockout of ACSL4 provided greater protection than knock out of LPCAT3 in inducible *Gpx4*^*-/*-^ murine embryonic fibroblasts (Pfa1 cells), which the authors suggest implies a more dominant role of ACSL4 in ferroptosis induction, however also demonstrates the redundancy of other LPCATs.^12^

Ferroptosis sensitive phospholipids can also be modulated to decrease ferroptosis risk. Phospholipase PLA2G6 (PNPLA9, iPLA2beta) metabolises hydroperoxide phosphatidylethanolamines to lyso-phosphatidylethanolamines and oxidized fatty acid, thus mitigating ferroptosis vulnerability.^59^ iPLA2beta genetic or pharmacological inactivation removes this layer of lipid peroxide defence and sensitises cells to ferroptosis.^60^ If not modulated to a ferroptosis resistant species, phospholipid hydroperoxides can also fracture into secondary products with short fatty acyl residues esterified in parental phospholipid (sn-2 position). These truncated oxidised phospholipids are structurally and functionally similar to platelet-activating factor (thus also known as PAF-like phospholipids), which has recently been shown to initiate and propagate ferroptosis.^61^ Ferroptosis could be suppressed by PAF-acetylhydrolase (II) (PAFAH2), another enzyme capable of modulating phospholipids by converting the short acyl chain into lyso-phospholipids, thus acting similar to iPLA2beta to remove oxidised lipids and protect against oxidative stress and ferroptosis risk.^61,62^

Evidence of the impact of phospholipid modulation on ferroptosis vulnerability has also been shown in cells treated with exogenous MUFAs.^63^ MUFA-treated cells displayed a ferroptosis resistant phenotype which was dependent on MUFA activation by acyl-coenzyme A synthetase long-chain family member 3 (ACSL3) and displacement of PUFAs from the plasma membrane. The protection was associated with a reduction in lipid reactive oxygen species and levels of phospholipids containing oxidizable PUFAs. Recently, the protection of MUFA treatment via phospholipid modulation has been confirmed in vitro.^64^ Mice fed a diet enriched in oleic acid (a MUFA) had reduced iron-overload induced liver lipid peroxidation and damage. Protection was associated with decreased levels of polyunsaturated fatty acyl phospholipids and ether-linked phospholipids.

Phospholipids can also be protected from autoxidation via 7-dehydrocholesterol, a cholesterol precursor synthesized by sterol C5-desaturase (SC5D) showing potent anti-ferroptotic activity.^65,66^ In the oncogenic environment, 7-dehydrocholesterol supports ferroptosis prevention by using the conjugated diene to prevent phospholipid autoxidation consequently protecting mitochondria and plasma membranes from phospholipid autoxidation and ferroptosis.^65,66^

## The role of iron in initiating ferroptosis

**Fig. 1:**
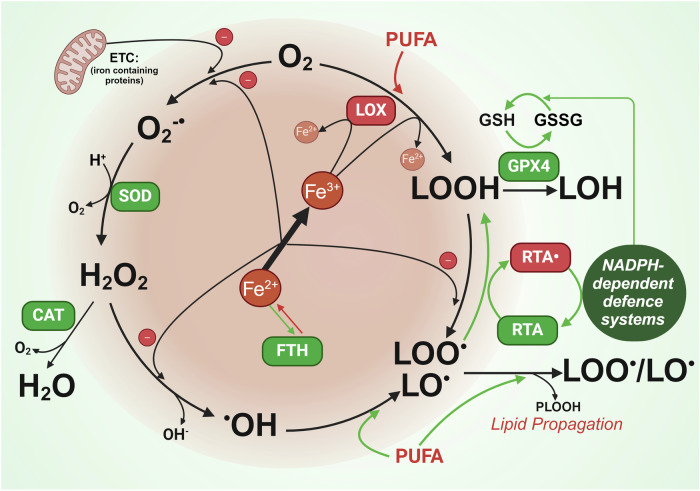
Central role of iron in reactive oxygen species generation and lipid peroxidation. Iron is involved directly and indirectly at several points to produce reactive oxygen species and lipid peroxidation. Indirectly, iron containing proteins in the electron transport chain (ETC) generate O_2_-• which is reduced to H_2_O_2_ by superoxide dismutase (SOD). H_2_O_2_ can either be quenched by catalase (CAT) or react with iron via the Fenton reaction, to generate hydroxyl radicals (•OH). The Fenton reaction can also catalyse the production of lipid peroxyl radicals (LOO• /LO•) from lipid hydroperoxide (LOOH). Radical trapping agents (RTAs) can quench lipid peroxyl radicals. Indirectly, iron contained in lipoxygenases (LOX) catalyse oxygenation of polyunsaturated fatty acids (PUFAs) and lipids to produce lipid hydroperoxide (LOOH). Glutathione peroxidase 4 (GPX4) can siphon lipid hydroperoxides away from fuelling lipid peroxidation and propagation by reducing PLOOH (high ferroptosis risk) to benign lipid alcohols (LOH). The reducing power of GPX4 is fuelled by reduced glutathione (GSH) which is dependent on NADPH to be recycled from its reduced form glutathione disulfide (GSSG). The breakdown of iron storage protein ferritin (FTH) can result in increased labile iron to facilitate these reactions. Figure created using Biorender.coms

Iron is an abundant metal on earth that almost all lifeforms depend upon.^67^ This transition element is important for a plethora of biological functions due to its capacity to redox cycle in two oxidation states within physiological parameters, Fe^2+^ and Fe^3+^, which enables the delivery and storage of oxygen, acid-base reactions and the conduction of electrons in the electron transfer chain.^68^ This same iron chemistry that biology exploits for a host of cellular functions also inadvertently causes oxidative stress and lipid peroxidation (Fig. 1).

If iron homeostasis is not in balance, the unique chemical properties of free iron can hamper cellular functions, primarily through the generation of oxidative stress and lipid peroxidation.^2^ The term “ferroptosis” which incorporates reference to iron (“ferrum”, the latin word for iron) was named in 2012 by Stockwell’s group based on the characterised “iron dependent” modality of cell death.^1^ This was based on the concept of labile iron being a catalyst for lipid peroxidation via Fenton- and Haber-Weiss–like reactions, in which H_2_O_2_ is reductively cleaved by ferrous iron to produce hydroxyl radicals that are then able to abstract a labile hydrogen from PUFAs.^69^ Iron containing enzymes can also facilitate the formation of PLOOH. Non-heme iron-containing lipoxygenases (LOXs) can also generate PUFA lipid hydroperoxides.^57,70^ The pro-ferroptotic role of LOXs is further evidenced by studies showing that knockdown (via siRNAs) or pharmacological inhibition of LOXs renders cells resistant to ferroptosis.^4,71^ However, LOX inhibitors have been proven to be potent radical-trapping antioxidants that protect lipids from autoxidation thus questioning the extent of which LOXs induce ferroptosis. While LOXs may contribute to the load of LOOH within the cell and potentiate ferroptosis vulnerability,^72–74^ the involvement of LOXs in initiating ferroptosis is still unclear.

While NADPH fuels several anti-ferroptotic proteins, there are two iron containing NADPH-dependent enzymes that can contribute to lipid peroxidation: (i) Heme containing NADPH oxidases (NOXs) which transfer electrons from cytosolic NADPH during the production of ROS which promotes lipid peroxidation^1,75^ and (ii) NADPH-dependent cytochrome P450 oxidoreductase (POR) which enables membrane polyunsaturated phospholipid peroxidation.^76^

## Iron regulation for ferroptosis defence

**Fig. 2:**
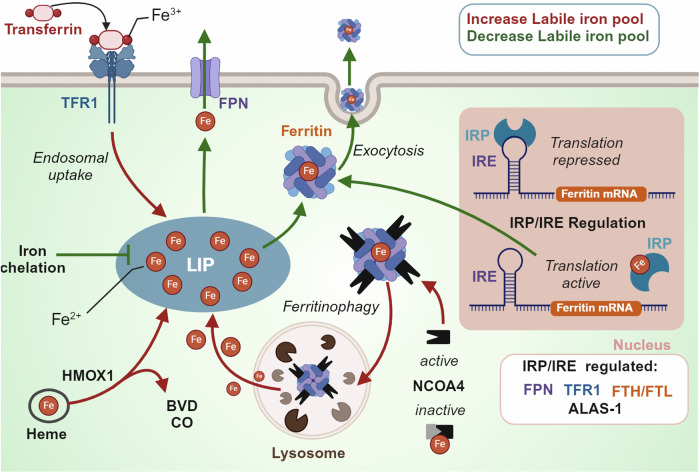
Cellular labile iron pool regulation. The labile iron pool is regulated by several proteins including i.) transferrin receptor 1 (TFR1) that facilitates iron influx in the form of transferrin, ii.) ferroportin (FPN) an iron channel facilitating iron export, iii) Ferritin which can store labile iron or release iron after lysosomal degradation which is mediated by nuclear receptor activator 4 (NCOA4) and/or iv.) Heme degradation by heme oxygenase 1 (HMOX-1) which releases iron and produces byproducts Biliverdin (BVD) and carbon monoxide (CO). The iron response protein/Iron response element (IRP/IRE) system responds to labile iron concentrations and subsequently regulates the expression of several proteins involved in iron regulation. Figure created using Biorender.com

To combat excess intracellular labile iron and consequently reduce ferroptosis vulnerability, the cell has two key defence strategies, 1. Sequester iron in ferritin: a “safe” non-toxic storage protein or 2. Control iron flux: Increase iron export and reduce import (Fig. 2).

### Ferritin and iron storage

The upregulation of ferritin to store labile iron mitigates free radical-mediated damage via labile iron and the Fenton reaction.^77–79^ There are two ferritin subunits, H and L, that facilitate iron detoxification and long-term storage in a redox-silent oxidised species.^80^ FTH1 has ferroxidase activity and converts reactive Fe^2+^ to a more stable Fe^3+^, which enables iron entry into the ferritin mineral core which is mediated by FTL.^81^ A single ferritin cage which consists of 24 H- or L- subunits can hold up to 4500 iron atoms.^82^

The iron response element/iron responsive protein (IRE/IRP) system is responsible for regulating the translation and subsequent expression of ferritin.^83,84^ The IRE/IRP system consists of a 5’ untranslated region of ferritin mRNA (IRE) and two RNA binding proteins (IRP1 and IRP2).^83,84^ IRP1 and IRP2 both respond to cellular iron levels but in different ways; IRP1 assembles an Fe-S cluster, turning it into an aconitase that cannot bind IREs, and IRP2 is targeted for proteasomal degradation by iron-dependent stabilisation of FBXL5, an E3 ligase that facilitates ubiquitination of IRP2.^85^ In both cases, increased iron leads to decreased IRE-binding capacity by IRP thus inhibiting the IRP from binding the IRE and repressing translation, resulting in increased ferritin synthesis along with other iron responsive proteins (i.e. ALAS-1).^86^ In contrast, under iron deprivation ferritin breakdown is activated by active nuclear receptor activator 4 (NCOA4) that binds and flags ferritin for lysosomal degradation and ferritin translation is repressed.^87^ Consequentially, degenerative models presenting with high tissue iron are often attempting to compensate with increased levels of ferritin.^88,89^

Autophagic degradation of iron storage processes also occur in response to ferroptosis induction.^90,91^ There are up to 35 autophagy related genes that contribute to the core autophagy machinery.^36,92^ Gao et al. used RNAi screening with genetic analysis to identify 11 autophagy related genes among other genes engaged in the pentose phosphate pathway and iron homeostasis as positive regulators of ferroptosis. Blockage of autophagy prevented the accumulation of labile iron and reactive oxygen species, thus preventing progression of ferroptotic cell death.^93^ Similarly, knockdown or knockout of autophagy related 5 and 7 (Atg5, Atg7) in fibroblasts and cancer cells decreased intracellular ferrous iron and lipid peroxidation in response to erastin, subsequently blunting ferroptotic cell death.^92^

The importance of iron sequestration and reduction is evidenced by attenuation of disease progression with either genetic (ferritin overexpression) or pharmacological modes (iron chelation).^90,91^ This is also true in the context of ferroptosis inducers where inhibition of NCOA4 prevented ferritin degradation and suppressed ferroptosis while overexpression of NCOA4 increased ferritin degradation and promoted ferroptosis.^36,92^ Other indirect ways of inhibiting ferritinophagy, such as via increased ApoE, have also shown protection against cysteine deficiency-induced ferroptosis.^19^ In addition to cytosolic ferritin, mitochondrial ferritin also exerts protection against erastin-induced ferroptosis.^94^

### Iron efflux and influx: transferrin receptor 1 and ferroportin

Iron influx is largely controlled by the expression of the Transferrin Receptor (TFRC/TFR1), a dimeric glycoprotein receptor for iron-loaded transferrin in the plasma. Transferrin binds to iron in the blood in a tight but reversible configuration and transports it systemically.^95^ TFRC extracellular domains have high affinity for iron-loaded transferrin to form a TF-TFRC complex which is internalized via receptor-mediated endocytosis.^95^ Iron is reduced in the endosome from Fe^3+^ to Fe^2+^ by the NAD(P)H-dependent transmembrane ferrireductase STEAP3 or by intraluminal ascorbate and released into cytosol via solute carrier family 11 member 2 (SLC11A2/DMT1).^95–98^ Subsequently, TFRC and TF are recycled back to the cell membrane and extracellular fluid, respectively. The dependence of iron delivery by transferrin and transferrin receptor was confirmed in a study that investigated serum factors that induced ferroptosis: both transferrin and transferrin receptor were required for serum dependent ferroptosis.^99^

Stockwell’s group conducted an antibody screen to detect ferroptosis in mice immunised with erastin treated membranes from lymphoma cells.^100^ Interestingly, they identified an antibody (3F3 ferroptotic membrane antibody) with a human transferrin receptor 1 protein antigen that was effective as a ferroptosis staining reagent, leading to the proposal that transferrin receptor is a selective ferroptosis indicator. Indeed, under some pathological conditions, cells that are susceptible to ferroptosis have an upregulation of TFR1 and down regulation of ferritin.^2,101^ In contrast, downregulation of TFRC has been shown to attenuate the ferroptosis by reducing iron import.^101^

There is only one known transmembrane exporter of non-heme iron, Ferroportin (SLC40A1/ferroportin/FPN1).^84^ Erastin induces the downregulation of ferroportin, which is prevented by ferroptosis inhibitors (iron chelation, ferrostatin-1 and N-acetyl cysteine).^102^ Ferroportin knockdown exacerbates erastin-induced ferroptosis, whereas genetic or pharmacological overexpression renders protection.^102,103^ In vivo, ferroportin surface expression is dictated by hepcidin, a protein secreted primarily by hepatocytes into the circulation where it binds to its receptor ferroportin causing its internalisation and degradation.^104^ Hepcidin is regulated by several factors including i.) HFE, a MHC class I-like protein that binds beta-2 microglobulin and TFRC in its extracellular α1-α2 domain,^105–107^ ii.) hemojuvelin (HJV), a membrane protein that acts as a co-receptor for bone morphogenetic protein (BMP) to signal via the SMAD pathway to regulate hepcidin expression^108,109^ and iii.) transferrin receptor 2 (TfR2), which acts as an iron sensor that can bind iron-loaded transferrin in the bloodstream, and hepatocytes leading to hepcidin upregulation.^110,111^ Mutations in these key hepcidin regulating genes that leads to a reduced production of hepcidin, or mutations in hepcidin and ferroportin, can lead to an iron overload disorder called hereditary hemochromatosis (HH). If left untreated, hemochromatosis leads to iron accumulation in the skeletal muscle, liver, heart, pancreas, and joints leading to fatigue, cirrhosis, arrhythmias, diabetes, and arthritis.^112–116^ Due to pathological iron overload, ferroptosis has been implicated as a mechanism of HH complications. In two hemochromatosis mouse models that develop severe iron overload (Hjv–/– and Smad4^Alb/Alb^ mice), elevated liver iron was associated with increased lipid peroxidation (MDA), decreased NADPH and liver damage that was attenuated with ferrostatin-1 treatment.^24^ This study also conducted microarray analyses of iron-treated bone marrow–derived macrophages and identified Slc7a11 as a candidate gene of ferroptosis in hemochromatosis, however future studies are required to characterise ferroptosis vulnerability in the human hemochromatosis population.

Exosomal transport of ferritin is a non-canonical cellular mechanism for exporting iron.^117,118^ Mammary epithelial and breast carcinoma cells survive in response to pharmacological and physiological ferroptotic stress due to an upregulation of a pathway involving multivesicular body/exosome expulsion of ferritin and iron out of the cell.^118^ This was shown to be mediated by the pentaspanin protein prominin2, which facilitated ferroptosis resistance via the formation of ferritin containing exosomes.^118^ Importantly, this mechanism introduced the concept of rapid modulation of intracellular iron levels ( < 2 h). Thus, controlling the labile iron pool via iron flux and storage is central to influencing ferroptosis susceptibility. However, some cancer cells that are reprogrammed to rapidly import iron for rapid proliferation are paradoxically resistant to ferroptosis.^119^ This is due to an additional layer of ferroptosis defence dictated by several systems utilising enzyme and metabolite coupling.

## Iron mediated ferroptosis defence in infection, inflammation and immunity

Iron dysregulation has recently been implicated as an initiating factor of ferroptosis in a range of different infectious diseases.^120–122^ Fundamentally, iron is a necessary element for successful infection.^123–125^ As a response, host defence mechanisms activated during infection attempt to restrict iron from pathogens. Mucosal surfaces that act as an entry point to many pathogens are coated with a fine layer of fluid that contains a high concentration of lactoferrin and lipocalin 2 that sequester iron to restrict the abundance of iron to microbes.^126,127^ Lactoferrin is structurally and functionally similar to transferrin as an iron transport molecule, however unlike transferrin that releases iron in acidified endosomes ( < pH 5.5), lactoferrin does not release iron even at a low pH (i.e., pH of 3.5), ensuring that iron restriction occurs in infected tissues that are often characterised by a highly acidic environment.^128,129^ Lipocalin-2 (pseudonyms; siderocalin or NGAL for neutrophil gelatinase-associated lipocalin) is secreted in humans and mice by epithelia, activated neutrophils and macrophages, to confiscate bacterial siderophores including enterobactin (secreted by a subset of E.coli and other Gram-negative bacteria) that bind ferric iron, thus sequestering ferric iron from the invading bacteria.^130,131^ A lack of lipocalin-2, conceivably a first line iron chelation defence against ferroptosis, increases the mortality in mice during E.coli sepsis or pneumonia.^132,133^

In response to infection or inflammatory stimuli, a cytokine-driven increase in hepcidin results in a drop of plasma iron, a response known as ‘hypoferremia of inflammation’.^129,134^ A drop in serum iron has been reported in several diseases associated with ferroptosis including Alzheimer’s disease,^135^ Parkinson’s disease^136^ and multiple sclerosis^137^ (discussed in depth later). Hepcidin downregulates ferroportin, thus decreasing iron export from cells. This is particularly beneficial for preventing the release from macrophages that actively collect and recycle iron. In hepcidin KO mice, hypoferremia of inflammation is absent or significantly reduced.^138,139^ Iron overload disorders (i.e., hereditary hemochromatosis or β-thalassemia), compromise host induced iron restriction due to impaired hepcidin action, and subsequently cause increased susceptibility to infections with microbes that can exploit this weakness.^129^ Hepcidin mutation is one cause of familial hemochromatosis.^140^ Beta-thalassemia suppresses hepcidin production due to an over population of erythroid precursors that release erythroferrone,^141^ a hormone that inhibits hepcidin transcription by inhibiting bone morphogenetic protein signalling in hepatocytes.^142,143^

Amaral et al. found that *M. tuberculosis* increased both labile iron and lipid peroxidation in infected macrophages.^120,144^ Initially described as necroptosis, the dying macrophages displayed a clear ferroptosis signature of high oxidised lipids and low GPX4 expression. Since cell death was also prevented by ferrostatin-1 or iron chelation, Amaral et al. redefined the cell death as ferroptosis. *M. tuberculosis* has also been shown to promote dissemination of ferroptosis by the secretion of protein tyrosine phosphatase A, which enters a host cell nucleus to promote asymmetric dimethylation of histone H3 arginine 2 via targeting protein arginine methyltransferase 6 leading to the inhibition of GPX4 expression.^145,146^ While key findings have been replicated in a mouse model of tuberculosis,^144^ the translation to human tuberculosis remains to be investigated. Due to the iron-scavenging properties of macrophages, they are also inherently vulnerable to ferroptosis (reviewed elsewhere^147^). The ferroptosis inhibitor ferrostatin-1 is reported to reduce cell death in ferric citrate challenged bone marrow-derived macrophages^24^ and to mitigate erythrophagocytosis in red pulp macrophages from a rodent model of transfusion.^148^

GPX4 expression is essential for the function of a range of different immune cells including CD8^+^ and CD4^+^ T cells, which fail to expand and protect against acute lymphocytic choriomeningitis virus and *Leishmania major* parasite infections when lacking Gpx4.^149^ Dendritic cells fail to secrete pro-inflammatory cytokines (TNF and IL6) and express MHC class I in response to the maturation signal of lipopolysaccharide upon GPX4 inhibition by RSL3.^150^

While not a focus of this review about ferroptosis defence, the immune response can also act to induce ferroptosis in pathological cells. CD8^+^ T cells or natural killer cells, key regulators of antitumor host immunity, release IFNγ, which has been shown to exaggerate glutathione depletion, lower mRNA and protein levels of two subunits of system xc^−^ (SLC3A2 and SLC7A11), and increase lipid peroxidation, so increasing sensitivity to ferroptosis activators.^151^ Ferroptosis in cancer cells is accompanied with elevated expression of PTGS2 and the release of prostaglandin E(2),^152^ which when certain levels are reached, play an immunosuppressive response.^153^ In addition, cancer cells dying from ferroptosis, in contrast to necroptosis, have also been shown to impede subsequent dendritic anti-tumour mechanisms.^154^ Thus, cancer cells may counteract ferroptosis with immunomodulation to progress tumour growth.^155^

## Enzyme metabolite coupling in ferroptosis defence

**Fig. 3:**
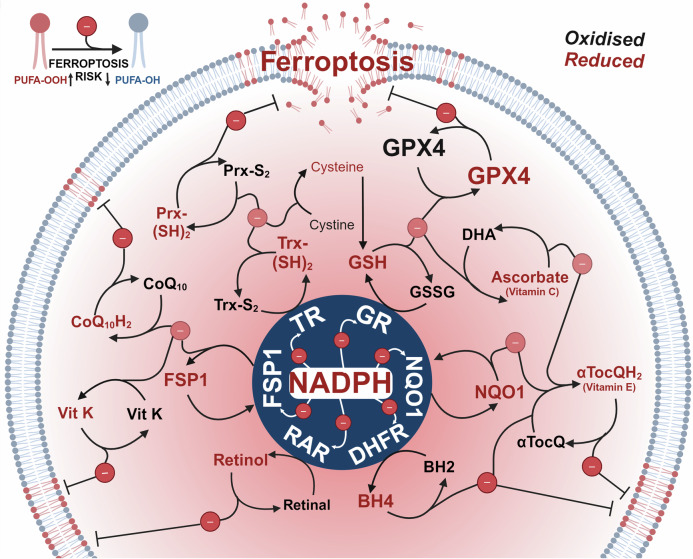
The reducing power of NADPH fuels ferroptosis defence. Each nicotinamide adenine dinucleotide phosphate (NADPH) molecule can donate two electrons. Electrons donated by NADPH reduce key anti-ferroptotic enzymes; glutathione reductase (GR), Ferroptosis suppressor protein 1 (FSP1), NAD(P)H Quinone Dehydrogenase 1 (NQO1), Dihydrofolate reductase (DHFR) and retinal dehydrogenases (RDH) and thioredoxin reductases (TR), which enable them to further propagate reduction reactions of multiple metabolites and proteins; retinol, retinal, tetrahydrobiopterin (BH4), dihydrobiopterin (BH2), α-tocopherol quinone (αTocQ), α-tocopherol quinol (αTocQH2), ascorbate, dehydroascorbate (DHA), glutathione (GSH), glutathione disulfide (GSSG), glutathione peroxidase 4 (GPX4), thioredoxin oxidised (Trx-S_2_), thioredoxin reduced (Trx-(SH)_2_), peroxiredoxin oxidised (Prx-S_2_), peroxiredoxin reduced (Prx-(SH)_2_), coenzyme Q10 (CoQ_10_), coenzyme Q10 reduced (CoQ_10_H_2_) and vitamin K (vit K), ultimately resulting in the prevention of lipid peroxidation. Figure created using Biorender.com

Several antioxidant systems target upstream and downstream pathways of lipid peroxidation. Antioxidant systems involve a complex interaction between reducing agents that can be proteins, metabolites, or vitamins. Several studies have investigated the implication of vitamin supplementation on ferroptosis; however, we will discuss the role of key vitamins (vitamin A, E, K and C) in their respective enzyme-metabolite coupling. A common thread uniting all key enzymes and metabolites that combat lipid peroxidation during ferroptosis is a dependence on a key cellular reductant, nicotinamide adenine dinucleotide phosphate (NADPH). Here, we will review the key ferroptotic defence enzymes and metabolites that depend on NADPH; 1.) glutathione reductase (GR), 2.) Ferroptosis suppressor protein 1 (FSP1), 3.) NAD(P)H Quinone Dehydrogenase 1 (NQO1), 4.) Dihydrofolate reductase (DHFR), 5.) retinal reductases and 6.) thioredoxin reductases (TR) (Fig. 3).

### Glutathione reductase (GR)/GSH/GPX4 and ascorbate (vitamin C)

Glutathione reductase (GR) is a key enzyme that replenishes reduced glutathione from the oxidised form. GR contains several highly conserved domains, one of which binds NADPH (residues 198–238).^156^ GR transfers two electrons from NADPH to GSSG, which results in the formation of two molecules of GSH and NADP + . GSH, a tripeptide composed of glutamate, cysteine, and glycine, subsequently reduces and recycles both 1.) GPX4 and 2.) ascorbate, along with a range of other metabolites and enzymes.

#### GSH/GPX4

As an initial electron donor, NADPH provides reducing power for the reaction and subsequent activation of major ferroptotic defence enzyme, GPX4.^152^ GPX4 is a selenoenzyme that prevents ferroptosis by detoxifying lipid hydroperoxides in cell membranes.^7,9,15^ The reducing power of GPX4 enables the reduction of PLOOH (high ferroptosis risk) to benign phospholipid alcohols (PLOH). GPX4 activity depends on the availability of reduced glutathione (GSH).^8^ Cysteine is considered the rate limiting substrate for GSH biosynthesis and hence sustained GPX4 activity.^157^ Circulating cysteine in the blood exists as the oxidised di-sulfide, cystine.^158^ Once imported via the system Xc- cystine/glutamate antiporter, NADPH catalyses the two-electron reduction of cystine to cysteine via thioredoxin reductase 1 (TXNRD1).^159^ Hence the maintenance of active GPX4 depends on NADPH at two levels to reduce both cystine to cysteine and to reduce GSSG to GSH.

GPX4 is regulated at transcriptional, translational and post translational levels. At a translational level, selenocysteine incorporation in the GPX4 active site is required to facilitate its protective function.^160^ Selenocysteine is a selenium containing amino acid enabling an oxidoreductase property in half of all selenoproteins.^161^ Selenium was initially regarded as a toxin present in agricultural feed,^162^ however, the tissue-protective function of selenium in ‘factor 3’ was soon appreciated in a rat model of liver necrosis (later characterised as ferroptosis^163^) due to dietary vitamin E deficiency.^164^ Selenium enacts a protective role as the amino acid selenocysteine (Sec), a crucial amino acid giving rise to an oxidoreductase property in half of all selenoproteins.^161^ The first selenoprotein discovered in mammals was glutathione peroxidase 1 (GPX1) which was thought to explain selenium deficiency induced peroxidation of unsaturated lipids in membranes.^165^ However, it was later confirmed that only GPX4 harbors the unique capacity to detoxify membrane lipid peroxides.^9,152^ The importance of selenium in ferroptosis defence was recognised due to its incorporation in GPX4.^160,166^ Conrad’s group have extensively explored the essential role of sec in Gpx4 through attempts to rescue the embryonic lethal Gpx4-/-mice.^166^ They show that a selenocysteine (Sec) to serine replacement in GPX4 does not protect from early embryonic lethality,^160^ and animals where a Sec is substituted to Cys (Sec differs from cys only by the substitution of sulphur for Se) in GPX4 fail to survive past 3 weeks.^160^

At a post translational level, ubiquitination/deubiquitylation and acetylation/deacetylation can regulate GPX4 activity and/or stability.^167–170^ For example, OUT deubiquitinase 5 (OTUD5) can bind and stabilise GPX4 thus preventing ferroptosis vulnerability, but MTORC1 activation induces autophagy and degradation of OTUD5 and consequently GPX4 decay and increased ferroptosis.^171^ Supraphysiological levels of the essential metal copper may also a role in GPX4 breakdown. Cu^2+^ can directly bind to GPX4 and induce the formation of GPX4 aggregates. This might account for GPX4 autophagic degradation mediated by Tax1 binding protein 1 in Cu^2+^-treated cells.^172^ A caveat in this report in that Cu^2+^ is not reported in the cytoplasm of cells under physiological conditions, where copper is believed to be only in the Cu^+^ oxidation state. However, copper chelators have also been shown to decrease ferroptosis vulnerability against erastin and RSL3 in vitro. In addition, copper treatment accelerated ferroptosis-induced tumour suppression in a mouse model of pancreatic cancer, which was associated with decreased expression of GPX4.^172^ Copper induced GPX4 deficiency may be relevant to copper overload conditions like Wilson’s Disease, an autosomal recessive genetic disease (mutation of ATP7B) characterized by copper overload and degeneration in multiple organs including the liver and brain. Recent studies in a copper loaded rat model of Wilsons Disease demonstrated decreased GPX4 expression and increased oxidative stress and lipid peroxidation markers, thus implicating ferroptosis as a potential mechanism underlying the neurological symptoms of Wilsons disease.^173^ Further studies are required to investigate the presence of GPX4 deficiency in human diseases. We hypothesise that common physiological ferroptosis defence limitations are more likely to manifest as NADPH deficiency, subsequently reducing GPX4 recycling, however rare GPX4 mutations that impact function and/or stability are known to exacerbate human disease pathology,^174,175^ possibly due to altered ferroptosis vulnerability.

#### GSH/ascorbate (vitamin C)

Both ascorbate (vitamin C) and GSH are abundant and stable antioxidants capable of donating electrons and scavenging various species of ROS. Unlike GSH which is synthesised intracellularly, ascorbate is acquired solely through the diet in humans, with severe deficiency leading to scurvy.^176^ Vitamin C exists in several redox states, including ascorbic acid/ascorbate and its two-electron oxidized form dehydroascorbic acid (DHA). DHA is reduced spontaneously by glutathione or enzymatically in reactions using glutathione or NADPH.^177^ Vitamin C and GSH can also directly interact with each other to exert a protective effect: glutathione can reduce oxidised vitamin C products through the glutathione-ascorbic acid cycle, thus shielding vitamin C from oxidation.^178–181^

In the oxidative stress context, reduced Vitamin C (i.e., ascorbate; regenerated by GSH and NADPH) directly reduces the tocopheroxyl radical (Toc•) to produce reduced tocopherol (Toc), which allows Toc to exert an anti-oxidant effect in lipid environments.^182^ As a direct anti-oxidant, studies have suggested that GSH and ascorbate have a partial redundancy in defence; in cells (human myeloid HL-60) with depleted GSH, pre- loading with vitamin C protected cells from death induced by H_2_O_2_.^183^ Conversely, the pharmacological enhancement of GSH by glutathione monoethylester can delay the onset of scurvy in rodents,^184^ probably via increased ascorbate stabilisation. However, the greatest reduction of ROS occurs when both ascorbate and GSH are present.^183^ In the ferroptosis context, where ascorbate cannot compensate for a lack of cysteine, vitamin C has been positioned as a ferroptosis inducer,^185^ since vitamin C can also act as a pro-oxidant. Under conditions of high ascorbate, vitamin C catalyses the reduction of free transition metal ions, like iron, which can cause the formation of radicals.^186^ In the few studies investigating the role of vitamin C in the context of ferroptosis, the pro-oxidant role of vitamin C was shown to predominate over its antioxidant capacity.^185,187,188^ Vitamin C supplementation induced lipid peroxidation, ROS and cell death associated with an inactivation of GPX4 that was partially rescued by DFO.^185,187^ In addition, increased vitamin C import via upregulation of SVCT2 promoted the reduction of intracellular Fe^3+^ to Fe^2+^, which reacted with excessive Vitamin C to produce severe oxidative stress and trigger ferroptosis in melanoma.^188^

### Ferroptosis suppressor protein 1 (FSP1), coenzyme-Q10, vitamin E and vitamin K

Apoptosis-inducing factor mitochondria-associated 2 (AIFM2) was initially identified as a pro-apoptotic gene,^189^ but it was later given another name ferroptosis suppressor protein 1 (FSP1) due to its newly appreciated role in ferroptosis defence.^190^ In the absence of functional GPX4, FSP1 defends against lipid peroxidation via 1) the NAD(P)H-dependent reduction of coenzyme-Q10 (ubiquinone) to the lipid peroxyl radical-quenching molecule, CoQ10-H2 (ubiquinol)^190,191^; and/or 2) the recruitment of endosomal sorting complexes required for transport (ESCRT)-III that repair oxidatively damaged sections of the plasma membrane.^192^ FSP1-mediated reduction of lipid peroxides is an alternative pathway to GPX4, but FSP1 and GPX4 are not redundant as their activities are differentially regulated and they act co-operatively.^190,191^ FSP1 can also prevent ferroptosis defence through the recycling of vitamin E and K.^190,191,193,194^ The investigation of FSP1 inhibitors recently lead to the discovery of a compound class of 3-phenylquinazolinones that induce phase separation of FSP1 into molecular condensates that renders cells vulnerable to ferroptosis inducers.^20^

#### Coenzyme-Q10

Coenzyme Q (CoQ) is a hydrophobic lipid consisting of a redox active benzoquinone ring fused to a polyprenoid tail of varying lengths of isoprenoid sidechains depending on the species (10 is the most common in humans, CoQ10).^195^ CoQ10 is ubiquitous in human tissue where it is manufactured at the mitochondrial inner membrane (IM).^196,197^ Due to a primary role as an electron carrier molecule in the electron transport chain to facilitate ATP production,^198^ CoQ10 is highly abundant in metabolically active tissue (ie., heart, liver, kidney and brain).^195^ However, reduced CoQ10 (ubiquinol) also acts as a potent antioxidant that traps lipid peroxyl radicals, consequently preventing ferroptosis.^190,191^ Oxidised CoQ10 in the cytosol is recycled by FSP1 using NAD(P)H acting as a glutathione independent system to suppress ferroptosis.^190,191^ Removal of CoQ10 from cells by blocking CoQ10 synthesis enzyme COQ2 lead to increased basal and RSL3 mediated lipid peroxidation.^191^

#### Vitamin E

Vitamin E is a lipid-soluble antioxidant that encompasses a group of compounds, including α-, β-, and γ-tocopherol (Toc) which have different chroman rings. α-tocopherol is the most biologically active and well-studied form in humans.^199^ Intracellular reduced α-tocopherol can act as a direct inhibitor of lipid peroxide propagation by donating one electron to an alkylperoxyl radical (LOO•) resulting in the production of a tocopheroxyl radical (Toc•) and LOOH.^200^ This disrupts the propagation step of lipid peroxidation, suppressing the further production of LOOH and consequent ferroptosis. In contrast, GPX4 suppresses ferroptosis by reductively converting LOOH to LOH.^182^ Reduced α-tocopherol can also suppress pro-ferroptotic lipoxygenase activity thus reducing the generation of doubly- and triply oxygenated (15-hydroperoxy)-di-acylated PE species.^57,190^

There are two key pathways cells use to regenerate reduced α-tocopherol: 1.) FSP1 uses NADPH to reduce CoQ10 to CoQ10-H2 which subsequently recycles oxidised α-tocopherol,^190,191,193^ and 2.) as previously discussed, reduced Vitamin C (regenerated by GSH and NADPH) directly reduces the tocopheroxyl radical (Toc•) to produces reduced tocopherol (Toc).^182^

In addition, overoxidation of α-tocopherol can yield a distinct chemical entity, α-tocopherol quinone, which exists in the oxidised state but can be reduced to α-tocopherol quinol, a highly active lipid peroxyl quencher.^201^

#### Vitamin K

Vitamin K is a fat-soluble antioxidant initially identified and characterised in 1934 for its key role in blood coagulation,^202^ and more recently has been shown to play a role in ferroptosis defence.^203^ Vitamin K is a term used for a range of compounds that share a common structure of a 2-methyl-1,4-naphthoquinone core, also known as menadione. K3 is regarded as the simplest form, containing only the core and serves as an intermediate in human metabolism and is not obtained through the diet.^204^ After intestinal absorption, dietary sourced vitamin Ks (i.e., phylloquinone (vitamin K1) and menaquinones (vitamin K2)) are transported into the blood by lipoproteins.^205^ Cellular uptake of vitamin K is mediated via lipoprotein receptors.

A screen of naturally abundant vitamin compounds in GPX4 knock out mouse embryonic fibroblasts identified three forms of vitamin K— phylloquinone, menaquinone-4 (MK-4), and menadione that could prevent cell death triggered by TAM-induced GPX4 deletion.^206^ In several human cell and mouse models vitamin K compounds enacted ferroptosis defence via inhibiting lipid peroxidation.^203,206^ FSP1 reduced via NADPH was identified as a vitamin K reductase that reduces vitamin K to its hydroquinone (VKH2) to support ferroptosis suppression.^194^ In addition to FSP1, vitamin K epoxide reductase complex subunit 1 like 1 (VKORC1L1) can also reduce vitamin K to generate vitamin K hydroquinone.^207^ VKORC1L1 was initially identified in CRISPR-Cas9 knockout screens as a ferroptosis suppressor.^207^ Currently, the physiological reductant of VKORC1L1 is unknown.

### NAD(P)H quinone dehydrogenase 1 (NQO1)

NAD(P)H quinone dehydrogenase 1 (NQO1) is an intracellular, cytosolic enzyme which catalyses the two electron reduction of quinones and other compounds including quinones, nitroaromatic compounds, imidazoles, and iron ions.^208,209^ The enzymatic function is initiated by binding an FAD cofactor, which is reduced by NAD(P)H.^210^ In the context of ferroptosis defence, NQO1 can function in the plasma membrane to recycle forms of ubiquinone^211^ and vitamin E, including alpha-tocopherol quinone^212^ (discussed previously), and as a direct superoxide reductase at high levels.^213,214^

Genetic manipulation of NQO1 has produced varying results indicating a context specific effect on ferroptosis. Deletion of NQO1 in human bone osteosarcoma U2OS cells did not impact RSL3 sensitivity.^191^ However, when deleted in combination with FSP1, cells were more sensitive to RSL3 than cells only deficient for FSP1. NQO1 overexpression in FSP1 KO cells promoted minor protection to RSL3. In contrast, in neuronal SH-SY5Y cells, overexpression of NQO1 resulted in increased lipid peroxidation following treatment with RSL3 and erastin, while NQO1 knockdown protected cells against ferroptosis by lowering iron and lipid contents and increasing GPX4, xCT, and the GSH/GSSG system.^215^ The reason for altered iron homeostasis was not explored, however the authors hypothesised that it was due to decreased proliferation driven by degradation of c-fos. NQO1 has recently been shown to directly interact with unstructured DNA-binding domain of c-Fos, which inhibits its proteasome-mediated degradation. This induces CKS1 expression and control of cell cycle progression at the G2/M phase leading to cancer proliferation.^216^ Indeed, NQO1 is pleiotropic antioxidant enzyme and has also been shown to control the stability of multiple proteins including p53,^217,218^ p73,^219^ p33ING1b,^220^ and HIF-1α.^221^

### Dihydrofolate reductase (DHFR), Tetrahydrobiopterin (BH4) and vitamin E

Dihydrofolate reductase (DHFR) regenerates tetrahydrobiopterin (BH4) while consuming NAD(P)H, which can act alone or in synergy with vitamin E as an endogenous radical trapping agent that protects lipid membranes from autoxidation.^222^ CRISPR-Cas9 screens identified BH4 as a metabolic modifier of lipid peroxidation upon GPX4 inhibition but not cysteine depletion.^222^ In response to erastin (but not RSL3), downregulation of BH4 via knockdown of the first-rate limiting enzyme of BH4 synthesis, GTP cyclohydrolase-1 (GCH1), increased lipid peroxidation and intracellular ferrous iron resulting in decreased in colorectal cancer cell viability.^223^ Supplementation of BH4 was sufficient to rescue erastin induced ferroptosis in GCH1 knockdown cells. Acting upstream of BH4, methotrexate synergizes with GPX4 inhibition to induce ferroptosis by reducing DHFR’s function.^222^

### Retinol and derivatives

Retinol (vitamin A) is a lipid soluble micronutrient absorbed through dietary sources including retinyl esters and β-carotene. While retinoids participate in a variety of physiological functions including affecting the expression of genes that regulate cell proliferation, differentiation and death,^224,225^ the anti-ferroptotic function is proposed to be primarily due to a radical trapping capacity that directly interdicts lipid radicals.^226,227^ However, vitamin A also has a higher reactivity compared to endogenous esterified PUFAs towards lipid peroxidation via autoxidation (propagation) and can thus divert free radical chain reactions away from membrane phospholipids to prevent ferroptosis.^228^ Retinol is regenerated from retinal by retinal reductases, which require NADPH.

### TRX (thioredoxin) and PRDX6

The thioredoxin system consists of thioredoxins (TRX) and thioredoxin reductase (TRXRD). TRXRD uses electrons from NADPH to reduce oxidised thioredoxin (TRX), which can subsequently reduce cystine to cysteine, the rate-limiting substrate for GSH biosynthesis and, in turn, regulate GPX4 activity.^229,230^ Indeed, overexpression of Trx-1 in mice reversed decreases of GPX4 induced by toxins used to model Parkinson’s disease (1-methyl-4-phenyl-1,2,3,6-tetrahydropyridine/1-methyl-4-phenylpyridinium (MPP + )).^229^ In vitro experiments confirmed that GPX4 deficiency and toxicity induced by MPP+ is rescued by ferrostatin-1 or by Trx-1 overexpression, implying a role of Trx-1 in ferroptosis defence.^229^ TRX was initially identified as an extracellular protein but is now known to localise intracellularly in the cytoplasm, mitochondria and nucleus.^231,232^ Together with NADPH, the TRX system reduces disulfide bonds in target proteins (i.e, peroxiredoxin family), restoring their activity and shielding them from oxidative damage.^233^

Peroxiredoxin 6 (PRDX6) is a member of the peroxiredoxin family of antioxidant enzymes that plays a crucial role in the repair of cell membrane lipid peroxidation.^234^ PRDX6 is a trifunctional enzyme that exhibits both peroxidase, phospholipase A2 (PLA2) and lysophosphatidylcholine acyl transferase (LPCAT) activities.^235,236^ The peroxidase activity enables PRDX6 to reduce peroxides and ROS by utilizing reducing equivalents from GSH and the thioredoxin system. The peroxidase activity can reduce a range of substrates with various implications; reduction of short chain hydroperoxides such as H_2_O_2_ would avert the formation of reactive oxygen (ROS) that are involved in biogenesis of lipid peroxidation, whereas the reduction of PLOOHs (i.e., phosphatidylcholine hydroperoxide (PCOOH)) would enable a lipid membrane repair process.^236,237^

## NADPH – the key metabolite fuelling ferroptosis defence

**Fig. 4:**
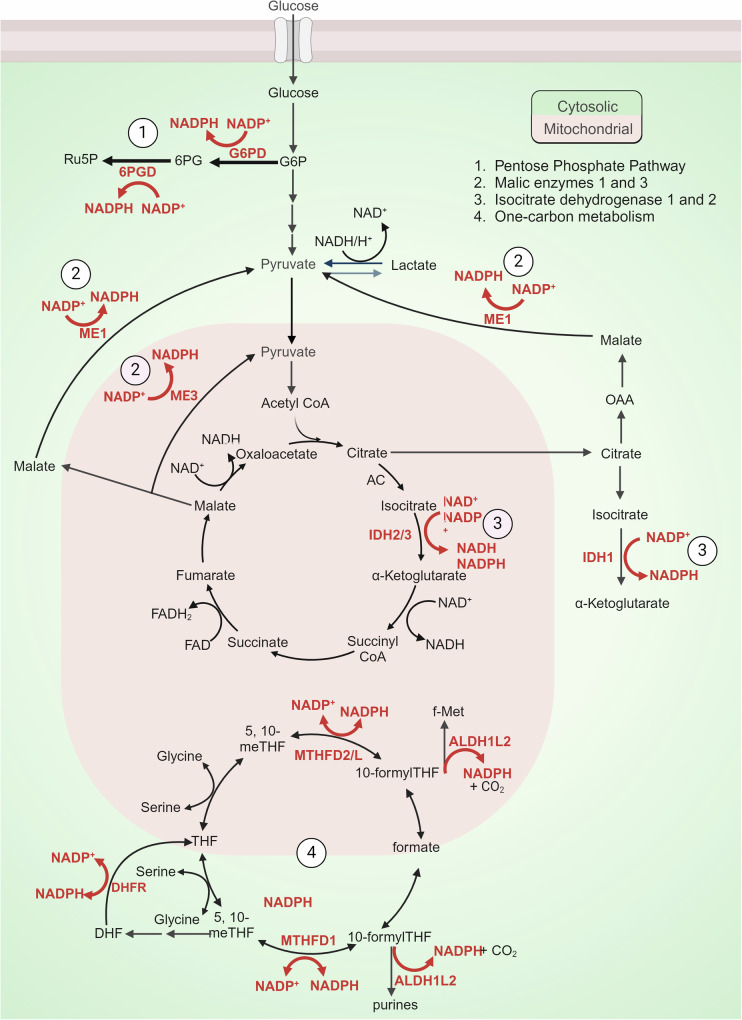
Key metabolic pathways fuelling NADPH generation. 1.) The pentose phosphate pathway, shunts from glucose-6-phosphate (G6P) to regenerate two nicotinamide adenine dinucleotide phosphates (NADPH) in two dehydrogenase steps i. G6P to 6-phosphogluconate (6PG) via glucose 6-phosphate dehydrogenase (G6PD) and ii. 6PG to ribose 5-phosphate (Ru5P) via 6 phosphogluconate dehydrogenase (6PGD). 2.) Malic enzymes 1, 2 and 3. Malic enzymes located within cytoplasm (ME1) and mitochondria (ME2 and ME3) catalyse the oxidative decarboxylation of malate to pyruvate while concurrently generating NADPH from NADP. 3.) Isocitrate dehydrogenases (IDHs) catalyse oxidative decarboxylation to produce NADPH. IDH1 localizes to varying extents to the cytoplasm, and IDH2/3 localise to the mitochondria. 4.) One-carbon (1 C) and folate metabolism which involves a series of 1 C transformations that generate and consume redox equivalents including the oxidisation of 10-Formyltetrahydrofolate (10-formyl-THF) to carbon dioxide (CO_2_) by cytosolic (1)/mitochondrial (2) 10-formyltetrahydrofolate dehydrogenase (ALDH1L1/2). NADPH can also be produced by reversible conversions of 5,10-methylene-tetrahydrofolate (5,10-meTHF) to 10-formylTHF by cytosolic (1) and mitochondrial (2) Methylenetetrahydrofolate Dehydrogenase (MTHFD1/2 L). Figure created using Biorender.com

NADPH is a substrate for both de novo lipid synthesis and for all key enzymes and metabolites that defend against ferroptosis, as the principal source of electrons (Fig. 3). NADPH also drives de novo synthesis of fatty acids, cholesterol, amino acids and nucleotides, as well as being used for nitric oxide signalling and ROS generation by NOX enzymes.^238^ To sustain these important roles in building biomass, signalling and cellular maintenance, the cell must constantly synthesise NADPH (Fig. 4).

### Metabolic pathways that produce NADPH

The oxidative pentose phosphate pathway is regarded as the major pathway for NADP^+^ reduction to NADPH and is a glucose-oxidising pathway, shunted from glucose-6-phosphate to produce ribose 5-phosphate via two dehydrogenase steps which regenerate two nicotinamide adenine dinucleotide phosphates (NADPH) (recently reviewed).^239^ The pentose phosphate pathway correlates with NADPH demand, which is enabled by NADP regulation of G6PD as a substrate and via an allosteric binding site on G6PD.^240,241^ Oxidative stress also imparts a higher NADPH demand and thus expression of several pentose phosphate genes (G6PD, 6PGD, TK and TALDO) are also upregulated by the nuclear respiratory factor 2 (NRF2) family of transcription factors.^242,243^ Genetic deficiencies in the pentose phosphate pathway occur commonly due to mutations in glucose-6-phopshate (discussed in more detail later).

NADPH can also be regenerated by cytosolic glycolytic and mitochondrial TCA cycle intermediates via Malic enzymes 1, 2 and 3. Malic enzymes, located in cytoplasm (ME1) and mitochondria (ME2 and ME3), catalyse the oxidative decarboxylation of malate to pyruvate while concurrently generating NADPH from NADP.^244,245^ Like the pentose phosphate pathway, MEs are upregulated in various cancer cell lines.^246,247^ Genomic deletion of ME2, which diminishes NADPH production, consequently induces higher levels of reactive oxygen species and cell death in pancreatic cancer cells.^247^

Isocitrate dehydrogenases (IDHs) are other enzymes that catalyse oxidative decarboxylation to produce NADPH. IDH1 and IDH2 share significant similarity and catalyse reversible reactions, whereas IDH3 catalyses an irreversible reaction with greater regulation (i.e., calcium, ADP and citrate), however all forms convert isocitrate to α-ketoglutarate while reducing NAD(P)+ to NAD(P)H.^248–251^ IDH1 localizes (variably) to the cytoplasm and IDH2/3 localise to the mitochondria.^252^ Examination of several gene expression databases from a range of cancer cell lines displayed co-expression of ME1 mRNA with G6PD and IDH1, indicating a coordination of metabolic pathways that produce NADPH.^246^

NADPH is also a product and substrate of several reactions in one-carbon (1 C) and folate metabolism, which involves a series of 1 C transformations that produce and consume redox equivalents.^245^ One of the only reactions that produces NADPH, and is not reversable, is the oxidation of 10-formyl-THF to CO_2_ by cytosolic (1)/mitochondrial (2) 10-formyltetrahydrofolate dehydrogenase (ALDH1L1/2).^253^ NADPH can also be produced by reversible conversions of 5,10-meTHF to 10-formylTHF, which is catalysed by methylenetetrahydrofolate dehydrogenases (MTHFDs).

The functional importance of diverse pathways leading to NADPH production is likely influenced by contextual factors including cell type and proliferative state. In HEK293T cells, to assess relative contributions of key pathways, the cellular NADPH/NADP+ ratio was measured after knockdown of a range of enzymes that produce NADPH.^245^ Malic enzyme 1 (ME1), cytosolic or mitochondrial NADP-dependent isocitrate dehydrogenase (IDH1 and IDH2) knockdown did not materially change NADPH/NADP + , however glucose-6-phosphate dehydrogenase or either isozyme of methylene tetrahydrofolate dehydrogenase (MTHFD1, cytosolic, or MTHFD2, mitochondrial) knockdown significantly lowered NADPH.^245^

### Other pathways involving NADPH

**Fig. 5:**
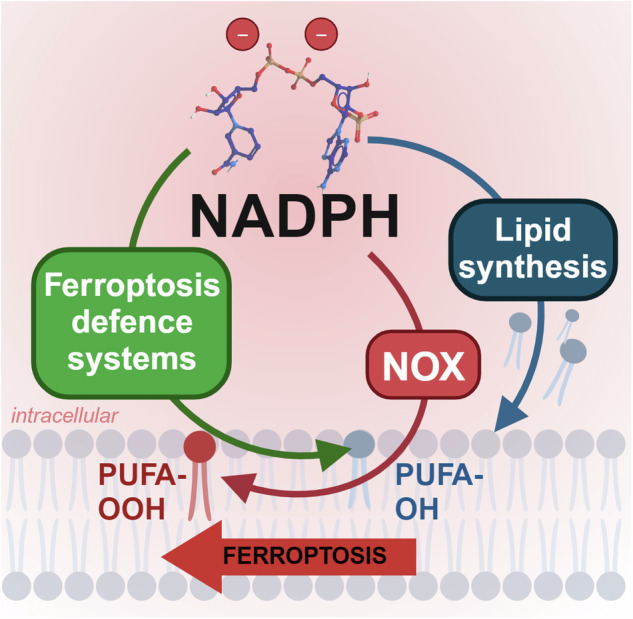
Dimorphic roles for NADPH in ferroptosis. NADPH promotes lipid synthesis for phospholipid production and is used by enzymes like heme-containing NADPH oxidases (NOXs) that transfer electrons from cytosolic NADPH to generate ROS, which promote lipid peroxidation (PUFA-OOH). Yet, NADPH is also recruited by anti-ferroptotic enzymes to prevent lipid peroxidation and to generate ferroptosis-resistant phospholipids (PUFA-OH). The recruitment of NADPH for ferroptosis-defence appears to be dominant in homeostasis, potentially to check the pro-ferroptosis pathways it fuels. Figure created using Biorender.com

While depleting NADPH sensitises to models of ferroptosis and oxidative stress,^254,255^ under certain contexts NADPH can promote the generation of substrates (i.e., ROS via NOX) for ferroptosis (Fig. 5). NADPH can donate electrons to the centre of NOX catalytic subunits to generate O_2_^-^ via the reduction of O_2_.^256^ Subsequently, SOD1 can convert NOX-generated O_2_^-^ to H_2_O_2._ NOX enzymes are important for a several biological functions including host defence, cellular signalling, stress response and transcription and translation regulation. NOX-generated ROS can be triggered by external environmental factors (e.g., hypoxia) and internal signalling (e.g., cytokines, hormones such as angiotensin II, aldosterone, endothelin-1, platelet-derived growth factor, transforming growth factor β and tumor necrosis factor α.^257–260^ Different members of the NOX protein family localise to specific membranes i) NOX1, -2, and -5 localised to the plasma membrane, ii) NOX4 is localised to the ER, mitochondrial and nuclear membranes.^256,261,262^

### Resources needed to synthesise NADPH

**Fig. 6:**
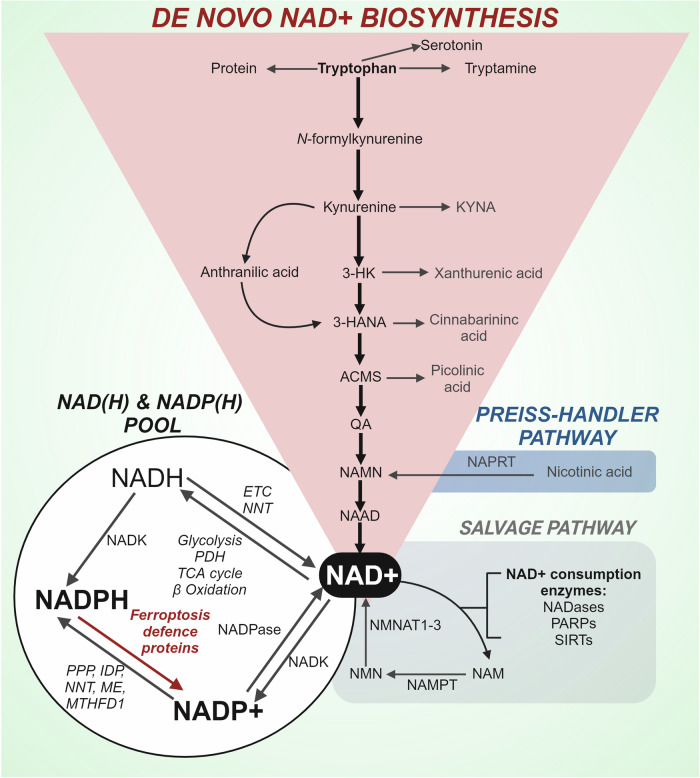
The NADP(H) pool and metabolism of NAD +. Mammalian cells use dietary tryptophan to synthesise nicotinic acid adenine dinucleotide (NAD + ) via the kynurenine pathway. The Kynurenine pathway has two key branches with the main path preferentially converting kynurenine into 3-hydroxykynurenine (3-HK) and then 3-hydroxyanthranilic acid (4-HANA), 2-amino 3-carboxymuconate 6-semialdehyde (ACMS), and quinolinic acid (QA), which is converted to nicotinic acid mononucleotide (NAMN), a common intermediate of the Preiss-Handler pathway. NAMN is subsequently metabolised to nicotinic acid adenine dinucleotide (NAAD) a direct precursor to NAD + . Several enzymes including NADases, Poly (ADP-ribose) polymerases (PARPs), and Sirtuins (SIRTs) utilise NAD+ as a substrate and generate nicotinamide (NAM). The salvage pathway regenerates NAD+ from the precursor NAM which is first converted by Nicotinamide phosphoribosyltransferase (NAMPT) to nicotinamide mononucleotide (NMN) and subsequently to NAD+ by Nicotinamide mononucleotide adenylyl transferase 1-3 (NMNAT 1-3). NAD+ contributes to the NAD(H) and NADP(H) pool via several metabolic pathways and enzymes; TCA cycle, tricarboxylic acid cycle; ETC, electron transport chain; NNT, nicotinamide nucleotide transhydrogenase; NADK, NAD kinase; PPP, pentose phosphate pathway; IDP, isocitrate dehydrogenase; ME, malic enzyme; MTHFD1, Methylenetetrahydrofolate Dehydrogenase; NAPRT, nicotinate phosphoribosyltransferase. Figure created using Biorender.com

NADP + , the oxidised form of NADPH, is formed by the phosphorylation of nicotinamide adenine dinucleotide (NAD + ) via NAD^+^ kinases (NADKs).^263^ Thus, maintaining the NAD^+^ pool is essential to facilitate NADPH production (Fig. 6). NAD^+^, an abundant metabolite in the human body, serves several functions including; a coenzyme for oxidoreductases, a substrate for several enzymes (sirtuin family deacetylases, poly (ADP)-ribosyl polymerases and cADP-ribose synthases), and a redox carrier for bioenergetic processes including glycolysis, the TCA cycle and fatty acid oxidation.^264^ As a result, NAD+ dysregulation is shared by different diseases (i.e., cancer,^265^ metabolic diseases^266^ and neurodegeneration).^267^

### De novo NAD+ biosynthesis

Mammalian cells can use dietary tryptophan to synthesise NAD^+^ via the kynurenine pathway. Despite being well known as a precursor for serotonin, over 95% of tryptophan is diverted to the kynurenine pathway.^268^ The rate limiting step of the kynurenine pathway (converting tryptophan to kynurenine) is facilitated by tryptophan dioxygenase (TDO) and indoleamine 2,3-dioxygenase (IDO).^269,270^ TDO is predominantly expressed in the liver whereas IDO is more broadly expressed, particularly abundant in cells of the immune and central nervous system.^271–273^ The kynurenine pathway has two key branches with the main path preferentially converting kynurenine into 3-hydroxykynurenine and then 3-hydroxyanthranilic acid and quinolinic acid, the latter of which is converted to nicotinamide mononucleotide (NAMN) and then to NAD^+^.^274^ Alternatively, kynurenine can be converted into kynurenic acid or anthranilic acid, with the latter feeding back into the main pathway via 3-hydroxyanthranilic acid. In addition to de novo biosynthesis, most cellular NAD^+^ is recycled via salvage pathways from nicotinamide (NAM), a by-product of NAD^+^ degradation.^275^

### NAD^+^ phosphorylation

The only way NADP^+^ is produced de novo is via phosphorylation of NAD^+^ by NAD^+^ kinases (NADKs) into NADP^+^ .^276^ This represents approximately 10% of the total NAD consumption.^277^ NADKs act via phosphorylating the 2’ position of the ribose ring connected to the adenine moiety. Since NADP^+^ and NADPH can not cross membranes, cells have subcellular localisation of NADKs; with NADK1 located in the cytosol and NADK2 in the mitochondria.^278^ NADP^+^ levels can be lowered via dephosphorylation by NADP phosphatase to produce NAD + .

### Low NADPH as a signature of ferroptosis

Demonstrating a central role for NADPH in ferroptosis, NADPH was shown to be depleted during ferroptosis of 60 cell lines, and cellular NADP(H) abundance predicted vulnerability to ferroptosis inducers.^10^ NADPH depletion occurs due to an imbalance of NADPH synthesis and hydrolysis (e.g., for usage in antioxidant defences during ferroptosis). The NADPH/NADP+ ratio favours the reduced form in the cytosol in physiological conditions.^263,279^ However, various stresses, diseases and pathological states that decrease NADPH, thus withdrawing the foundation of ferroptosis defence, may render cells susceptible to ferroptosis. Hence, the level of NADPH could be considered a biomarker for ferroptosis sensitivity.^280^

Human Metazoan SpoT Homologue 1 (MESH1) was recently identified as a NADPH phosphatase with its upregulation consequently depleting NADPH, resulting in an impairment of glutathione regeneration and increased ferroptosis.^255^ Conversely, MESH1 removal preserved the NADPH pool in stressed cells and promoted their ferroptosis resistance. Higher levels of NADPH have also been shown to correlate with greater resistance to ferroptosis in cancer cells.^10^ Cancer cells are known for metabolic reprogramming that diverts carbon flux towards anabolic pathways such as the pentose phosphate pathway (PPP) to enable both rapid proliferation and generation of NADPH.^239,281^

## Regulation of ferroptosis defence systems

Transcription factors can regulate gene expression programs that coordinate a parallel ferroptosis defence response through multiple pathways and organelles.^282^ The ER plays a crucial role in both lipid synthesis and the processing of transcripton factors that can initate or potentiate a ferroptosis defence gene expression program. Transcription factors in the ER including sterol regulatory element-binding proteins (SREBPs), regulate the expression of several enzymes involved in lipid metabolism (i.e., *ACLY*, *ACACA*, *FASN*, and *SCD)* and glucose metabolism including key proteins regulating the PPP (i.e., *PKLR*, *PCK1*, *G6PC*, and *G6PD)*.^283–286^ SREBPs are tethered to the ER membrane and when activated are trafficked to the Golgi where they are proteolytically processed to release an active transcription factor that is subsequently imported into the nucleus to initiate trascription. SREBP1 has been reported to be regulated by PI3K-AKT-mTOR signalling,^287,288^ one of the most commonly altered signalling pathways in human cancers.^289–291^ As a result, oncogenic activation of the PI3K-AKT-mTOR signaling pathway induces protection against ferroptosis and pharmacological inhibition of this pathway could induce vulnerability to ferroptosis induction in cancer cells.^292^ A study looking at the impact of SREBF1 knockout, identified reduced expression of Stearoyl-CoA Desaturase 1 (SCD1, both mRNA and protein levels) as the most significantly impacted target. SCD1 catalyzes the rate-limiting step in MUFA synthesis, and since MUFAs are not vulnerable to peroxidation, protects against ferroptosis.^292,293^ Inhibition of SCD1 decreased CoQ_10_, an endogenous membrane antioxidant previously discussed, induced lipid oxidation and exacerbated ferroptosis sensitivity.^294^

The Nuclear factor erythroid 2–related factor 2 (NRF2)/NFE2L2-Kelch–like ECH-associated protein 1 (KEAP1) pathway is another well-known master regulator of cellular defence against ferroptosis, which also regulates NADPH generation and consumption.^243,295,296^ In unstressed conditions, NRF2 is minimally detected due to a very rapid half-life (less than 20 min).^297^ Keap1 acts as both an anchor that inhibits Nrf2 nuclear import and an adaptor that facilitates binding with Cullin 3-based E3 ligase, a protein-protein complex that ubiquitinates Nrf2 protein and leads to its rapid degradation through the proteasome system.^298^ Human Keap1 has 27 cysteine residues (25 cysteine residues in murine and rat) which are modified by both NRF2 activating compounds or oxidative/electrophilic stress.^296,299,300^ Oxidation, reduction, or alkylation of the sulfhydryl groups of cysteines in KEAP1 alter the conformation leading to the release of NRF2.

Unbound NRF2 translocates into the nucleus before heterodimerizing with musculoaponeurotic fibrosarcoma (Maf) protein and promoting the transcription of over 200 phase II and antioxidant genes.^301–303^ NRF2-regulated genes that are involved in ferroptosis defence include FSP1, GPX4, and xCT (full list available in ref. ^304^). In addition to several anti-ferroptotic proteins previously described to consume NADPH, NRF2 also regulates proteins that regenerate NADPH, including G6PD,^296,305,306^ PGD^296,305^ and ME1.^296,305,307^ Nrf2-regulated NADPH generation and consumption was investigated in Nrf2-null mice and Keap1-knockdown mice, with the latter having a higher concentration of hepatic NADPH.^296^ The authors indicated that NRF2 may also indirectly regulate the consumption of NADPH by downregulating genes involved in fatty acid synthesis and desaturation, concluding that Nrf2 protects against oxidative/electrophilic stress by helping with the production of NADPH.

## The link between NADPH and ferroptosis in disease contexts

### G6PD deficiency

Glucose-6-phosphate dehydrogenase (G6PD) deficiency is the most frequent human enzyme defect,^308^ causing hemolytic anemia upon exposure to certain stresses like infection, fava beans, aspirin etc. As the catalyst in the rate-limiting first step of the PPP which produces NADPH, G6PD deficiency disrupts a major metabolic pathway required to produce NADPH and power anti-ferroptotic defence. Complete G6PD deficiency is embryonically lethal in mice, but the human G6PD gene has over 200 variants, with the majority being missense mutations resulting in an unstable G6PD enzyme and G6PD deficiency.^309^ This helps explain why G6PD deficiencies due to these mutations predominantly affect red blood cells: mature red blood cells lack the ability to synthesise new proteins so they cannot replace mutant G6PD, which is more unstable and has a shorter half-life.^310,311^ The G6PD/NADPH pathway is the sole source of reduced glutathione in red blood cells. Red cells are put at risk of ferroptosis because they carry high concentrations of oxygen and iron, and must heavily rely upon the protection of G6PD/NADPH/glutathione. Comparatively, nonerythroid organs can compensate with increased G6PD synthesis and have metabolic changes consistent with mild G6PD deficiency.^312^ Certain medications and external sources of oxidative stress (i.e. infection) exploit inherent vulnerability in G6PD deficient individuals, primarily due to a decreased capacity to produce NADPH.^313^ The most common clinical presentations include acute haemolytic anaemia and neonatal jaundice, but studies have suggested an increased prevalence of diabetes mellitus and kidney disease.^314^ The avoidance of oxidative stress is one of the most beneficial management strategies to prevent haemolysis in patients with G6PD deficiency.^308^ While ferroptosis has not been explicitly studied in the context of G6PD deficiency, it is likely that individuals with G6PD deficiency and consequent NADPH depletion are more vulnerable to ferroptosis.

In contrast, various studies have stated a reduced incidence and mortality for specific cancers with hypomorphic mutations in G6PD.^315–317^ Metabolic re-wiring to upregulate the PPP and NADPH is characteristic of many cancers to boost oxidative stress and ferroptotic defence and provide metabolites for nucleotide and lipid synthesis. A recent pan-cancer study promoting a theoretical basis for developing G6PD inhibitors as anti-cancer drugs confirmed increased G6PD expression in hepatocellular carcinoma, glioma and breast cancer.^281^ In addition, a search for synthetic-lethal genes for neurofibromatosis Type II, a genetic condition characterised by benign tumors of the peripheral nervous system, using a genome-wide CRISPR/Cas9 screen identified ACSL3 and G6PD as two lethal partners; which was partly attributed to a diminished expression of genes associated with NADPH abundance.^318^ Pentose phosphate pathway metabolites are also enriched in metastasizing melanomas to generate NADPH for oxidative stress resistance.^254^ When G6PD activity is impaired in patient-derived melanomas, via mutation of the substrate binding site, mutant melanomas experience increased oxidative stress and decreased NADPH and GSH which suggests an increased metabolic vulnerability to ferroptosis when the PPP is impaired.^254^

The relationship between cholesterol synthesis and ferroptosis has recently been investigated in cancer cells.^65,66^ Enzymes and metabolites implicated in distal cholesterol biosynthesis have contrasting roles in regulating ferroptosis with 7-dehydrocholesterol, a cholesterol precursor synthesized by sterol C5-desaturase (SC5D) showing potent anti-ferroptotic activity. Interestingly DHCR7, the key enzyme converting 7-dehydrocholesterol to cholesterol, thus reducing 7-dehydrocholesterol, is also dependent on NADPH. In the oncogenic environment, 7-dehydrocholesterol conducts ferroptosis surveillance by using the conjugated diene to prevent phospholipid autoxidation, consequently protecting plasma and mitochondria membranes from phospholipid autoxidation and ferroptosis.^65,66^ However, in a non-oncogenic context, 7-dehydrocholesterol itself is extremely prone to free radical autoxidation resulting in the production of a dozen different toxic oxysterols.^319^ In a study before ferroptosis was coined, high concentrations of 7-DHC-derived oxysterols were cytotoxic to developing neurons by encouraging lipid peroxidation.^320^ Future studies are required to investigate the role of DHCR7 in preventing ferroptosis outside the oncogenic environment, as it may be another fundamental ferroptosis defence enzyme dependent on NADPH.

## Ferroptosis in neurodegenerative contexts

### Stroke, infarction and Ischemia-reperfusion damage

Ischemia is due to a restriction of blood flow that limits both the replenishment of oxygen and nutrients and the elimination of metabolic wastes from affected tissues. The subsequent reperfusion is needed to retain tissue function and viability, but while essential to prevent hypoxic damage, reperfusion paradoxically introduces a second oxygen chemical lesion that exacerbates oxidative stress.^321^ This can occur in various tissues and organs including the heart, kidney and brain due to a reduction of blood flow by a physical obstruction of a vessel or by a deleterious redistribution of blood flow away from a tissue or organ. In the heart, myocardial infarction can be caused by coronary atherosclerosis or the rupture of an artery plaque, which can trigger thrombosis and artery occlusion.^322^ In the kidney, interruption of renal blood flow is the leading cause of perioperative acute kidney injury which can occur in several clinical settings including major surgeries, sepsis, trauma and transplantation.^323^ In the brain, reperfusion damage is common post-stroke (e.g. after clot retrieval or thrombolytic treatment).

Several studies suggest that ferroptosis as a key mechanism involved in the onset and progression of ischemic-reperfusion injury in a range of organs.^324,325^ Ferroptosis inhibitors liproxstatin-1^326^ and ferrostatin-1^327^ reduce cell death and infarct size while maintaining mitochondrial integrity in ischaemic-reperfusion heart injury models. Ferroptosis has also been implicated as a significant cell death pathway in ischemic reperfusion-induced acute kidney injury, particularly in renal tubular cell death, which is also alleviated with ferrostatin^328^ and liproxstatin.^329^ Interestingly, one model describes NADPH abundance as a gradient that defines the risk of ferroptosis and dictates progression of synchronized cell death in renal tubules.^330^ Briefly, an initiating cell under oxidative stress undergoes NADPH-depleting necrosis while it is linked to the cytoplasm of neighbouring cells via tight junctions and gap junctions that subsequently recruits NADPH from the adjacent cells. These neighbouring cells are therefore at risk for ferroptosis due to a depletion of NADPH. In ischemic stroke models, a model which are strongly associated with ferroptosis, iron elevation associated with ischemic damage is mitigated by ferroptosis inhibitors liproxstatin-1 and ferrostatin-1,^331^ selenium supplementation to enhance GPX4 activity,^332,333^ ceruloplasmin to facilitate iron export,^334,335^ or genetic mutations that decrease labile intracellular iron; tau-knockout mice,^336,337^ which increase iron export and NCOA4 deletion, prevent ferritinophagy.^334^

According to studies that suggest excessive lipid peroxidation and cell death begins in the ischemic period, pre-treatment with ferroptosis inhibitors DFO and ferrostatin-1 reduced cytotoxicity and reversed a depletion of total GSH and NADPH.^338^ This could imply that the abundance of NADPH promotes resistance to lipid peroxidation, indicating a role for NADPH in the defence against ferroptosis during ischemia. Indeed, NADPH supplementation increased ATP and the reduced form of glutathione, lowering intracellular oxidative stress and protecting neurons against ischemia/reperfusion-induced injury.^333^

Conversely, NADPH may also be utilized by NADPH oxidases (NOXs) to produce ROS in certain contexts.^339^ NADPH oxidase (Nox) 2 and 4 are the major sources of O^2−^ and H_2_O_2_ in the heart and are upregulated in response to ischemia-reperfusion.^340^ Suppression of either can reduce ROS and IR injury, but synergistic inhibition of both Nox2 and Nox4 exacerbates myocardial I/R injury.^340^ In a model of intermittent hypoxia, (ROS) generated by NADPH oxidases (specifically Nox2) created a signal necessary to increase HIF-1α synthesis and stability for metabolic adaptation.^341^ Thus, NADPH abundance plays a paradoxical role in both preventing and contributing to ferroptosis in ischemic reperfusion injuries.

Clearly the ability to re-wire central carbon metabolism in ischemia, and inevitably alter NAD(P)H levels, plays a key role in pathogenic hypoxia adaptation, however the direct implications and timing of NAD(P)H in ischemic-reperfusion induced ferroptosis requires further characterisation. Utilising novel tracer methods to measure metabolic flux during hypoxia will shed light on the contribution of NADPH to ischemia reperfusion-induced ferroptosis. While NADPH production is often attributed to PPP flux, to accurately characterise NADPH metabolism, studies should combine several tracers (deuterium (2H) tracer methods) to quantitatively analyse NADPH production from all potential pathways including the PPP, folate metabolism and malic enzymes.^342^ Future studies should subsequently investigate the function of NADPH produced during hypoxia and deliberate whether and how it contributes to ferroptosis initiation or defence.

### Alzheimer’s disease (AD)

AD is the most common dementia with late-onset sporadic AD accounting for 95% while familial AD caused by autosomal dominant mutations accounts for less than 1% of all diagnosed cases.^343^ The accumulation of amyloid-β (Aβ) plaques and neurofibrillary tangles composed of hyperphosphorylated tau are pathological hallmarks of AD. However, high clearance anti-amyloid therapies have recently been approved by the FDA, show modest efficacy in slowing cognitive deterioration, as well as serious adverse effects.^344,345^ Studies have also identified a potential ferroptosis signature in AD models consisting of disrupted iron homeostasis, decreased GSH and increased markers of lipid peroxidation and oxidative stress (recently reviewed^346^).

Before ferroptosis had been defined, iron accumulation was observed in AD brains and was implicated as a contributing factor to disease progression.^347^ Elevated CSF ferritin, a biomarker of brain iron, is associated with the Alzheimer’s major risk allele, APOE e4 and AD progression (cognitive decline and brain atrophy).^348–350^ An association with iron and AD progression was later confirmed in quantitative susceptibility mapping (QSM) –magnetic resonance imaging^351^ and directly in post-mortem brains.^352,353^ Unbiased ‘omics analyses have also identified iron homeostasis as a biological process affected in AD.^354,355^ Excessive lipid peroxidation is more direct evidence of ferroptosis. Indeed, multiple biomarkers of lipid peroxidation (including F2-IsoPs, 4-HNE, malondialdehyde, and protein-bound acrolein) are elevated in CSF, post-mortem AD brain samples and animal models of AD.^356,357^ Substituting dietary PUFAs with deuterium-reinforced PUFAs, which are more resistant to lipid peroxidation, supresses lipid peroxidation (cortex and hippocampus), and improves cognition in a model of oxidative stress-related cognitive impairment that exhibits AD-like pathologies.^357^

Recent studies report a decrease in GSH in the hippocampus, frontal cortex, and cingulate cortex of AD subjects^358–362^ and a positive correlation with impairment in Mini-Mental State Examination scores.^360^ Low GSH suggests that the AD brain is unable to maintain a strong defence against excessive lipid-peroxidation, potentially enabling ferroptosis. In a clinical study, levels of GSH in the left hippocampus of patients with mild cognitive impairment and AD were inversely correlated with iron.^358^ Oxidised GSH may serve as a proxy for a depletion of NADPH within the cytoplasm, however further studies are needed to unpack the metabolic dysregulation involved. An indirect way of assessing NADPH in AD is to measure the pentose phosphate pathway. Limitations of phosphometabolite detection have prevented a comprehensive metabolite assessment, however studies have looked at the key enzyme G6PDH. Evidence in AD patients is conflicting and restricted by small sample sizes, with a decrease in synaptosomal G6PDH activity observed in the frontal cortex^361^ and hippocampus,^363^ and an increase in the inferior temporal cortex^364^ and cerebral hemisphere.^365^ To add to the complexity, an upregulation of PPP to produce NADPH may not necessarily increase the availability of NADPH for ferroptosis defence systems since studies report a decrease in several NADPH-dependent antioxidant systems (GPX, CAT, and PRDX) and an increase in NAPH-dependent NOX activity.^361,366,367^ Moreover, an elevated NADPH/NADP+ ratio significantly reduces G6PD activity, thus proteomic studies cannot delineate a directionality of PPP regulation and subsequent NADPH generation.^368,369^

Other hallmarks of AD progression may also implicate an impairment of NADPH production due to depleted NAD + . Mitochondrial impairment is well-documented in AD pathogenesis and may be caused by complex I impairment and/or impaired lysosomal-dependent mitophagy.^370^ The mitochondrial electron transport chain contains several iron-containing proteins, and iron chelation can decrease mitochondrial energy production. Thus, we highlight caution when using iron chelation as an anti-ferroptotic strategy in diseases with underlying mitochondrial pathologies.^86,88,89,371^ As complex I is crucial to reduce NADH to NAD + , dysfunctional complex I or mitochondrial impairment may lead to a loss of NAD + . Supplementation with NAD+ precursors has been explored as a treatment for cognitive decline (recently reviewed^372^). Across multiple models of cognitive decline (Alzheimer’s disease, vascular dementia, diabetes, stroke, and traumatic brain injury), the majority report cognitive benefits,^373,374^ however others have reported null or adverse effects. In 2021 a systematic metanalysis in investigating NAD + , its derivatives, and their association with cognitive function restricted to the AD context revealed that NAD+ improves learning and memory.^374^ Subsequent studies concurred, showing cognitive benefits in a range of rodent AD models including APP/PS1-mutant mice,^373,375^ and intracerebroventricular injection of Aβ1–42.^376^

There are no clinical studies investigating NADPH and limited comprehensive and well controlled human studies on the impact of supplementation with NAD+ precursors on cognitive function in AD. In 1996, 17 Alzheimer’s disease patients were treated with NADH disodium salt (10 mg/day) which benefited cognitive function based on the MMSE.^377^ However, an attempt to repeat these findings in 2000 included non-Alzheimer’s dementia in their study and showed no effect.^378^ The inconsistency suggests that NAD+ precursor supplementation might be selectively beneficial for AD among dementias, where there is a metabolic dysregulation that depletes NADPH resources, potentially an energetic failure, leading to increased ferroptosis susceptibility.

### Parkinson’s disease

Parkinson’s disease (PD) is characterised by α-synuclein aggregation in cells of the midbrain dopaminergic neurons and cortical neurons. Chronic and progressive neurodegeneration of dopaminergic neurons results in the motor symptoms of PD including tremors, rigidity and bradykinesia. Early studies demonstrated iron elevation, excessive oxidative stress and damaged lipids in the most severely affected subpopulation of melanized neurons located in the substantia nigra pars compacta,^379–381^ implicating a potential role of ferroptosis. In addition, epigenetic modifications involving hypermethylation and consequently downregulation of system xc- has been shown to be associated with PD.^382^

Excess iron in PD brains has been attributed to various mechanisms including iron-loaded neurotoxic microglia,^383^ α-synuclein stabilisation of DMT1,^384^ α-synuclein binding with iron^385^ and amplified IRP1 activity resulting in a reduction of ferritin concentrations and increase in TfR1 expression,^3^ decreased transferrin and ceruloplasmin, which facilitates iron export from ferroportin and decreased APP, which stabilizes ferroportin at the cell surface.^91,386,387^ Aberrant α-synuclein oligomer incorporation into membranes of human iPSC-derived neurons with SNCA triplication, led to dysregulated membrane conductance, abnormal calcium influx and lipid peroxidation.^388^ Erastin exacerbated α-synuclein oligomer induced toxicity in human iPSC-derived neurons with SNCA (α-synuclein gene) triplication, which was reduced by three classes of ferroptosis inhibitor- deuterated PUFAs, iron chelator deferoxamine, and ferrostatin-1.^388^ Similarly, ferroptosis evasion occurred in neurons depleted of α-synuclein, which was attributed to a reduction in ether-linked phospholipids that are essential for ferroptosis.^389^

In addition to iron accumulation and excessive lipid peroxidation, further ferroptosis vulnerability in PD is demonstrated by a depleted glutathione levels, decreased system xc- and diminished coenzyme Q10.^390^ In a mouse model of PD, GPX4 levels were decreased in midbrain dopaminergic neurons.^391^ The proposed mechanism suggested that iron-induced dopamine oxidation modified GPX4 leading to its degradation. Conditional knockdown of Gpx4 in substantia nigra was also shown to accelerate the onset of parkinsonism in *SNCA*^A53T^/*Gpx4*^+/fl^ double transgenic mice.^391^ Several features of PD pathology parallel the ferroptosis pathway, opening therapeutic opportunities of targeting this cell death pathway discussed below.

Mitochondrial dysfunction, specifically a reduction in Complex I activity has also been characterised as a hallmark of PD.^392^ Disruption of mitochondrial complex I via deletion of Ndufs2 specifically from dopaminergic neuron downregulation in mice induced a Warburg-like metabolic shift (upregulation of genes associated with glycolysis and downregulation of those genes associated with OXPHOS) that enabled neuronal survival but triggered progressive, axon-first, levodopa-responsive parkinsonism^393^ as observed in humans.^394^ In rodents, the complex I inhibitor rotenone, has also been able to induce parkinsonism clinical, pathological, and biochemical characteristics.^395,396^ Mechanisms of toxicity in rotenone models of PD rule out a depletion of ATP, since glycolysis inhibitors deplete ATP to a similar magnitude as rotenone but do not cause toxicity.^396^ Instead, excessive oxidative stress and dopaminergic neuronal loss is blocked by α-tocopherol, a anti-ferroptotic nutrient. Thus, it is likely that excess oxidative stress exerts a tax on NADPH in neurons. In humans, dietary vitamin E has emerged as a protective factor of PD, where increased Vitamin E dietary consumption was inversely associated with PD occurrence irrespective of age and gender.^397^

### Huntington’s disease

Huntington’s disease (HD) is an autosomal dominant progressive and ultimately fatal neurodegenerative disease caused by an expanded CAG repeat in the huntingtin gene.^398^ The toxic gain of function has been attributed to a polyglutamine strand of variable length at the N-terminus which leads to misfolding and protein aggregate formations. Mutant huntingtin aggregates affect a variety of cellular functions, however the precise mechanism leading to neuronal cell death is poorly understood.^399^ Ferroptosis has been implicated due to the classic ferroptotic signature of increased iron accumulation and lipid peroxidation with decreased GSH and blunted NRF2 response.^400–404^ Anti-ferroptotic compounds have also shown efficacy; ferrostatin-1 prevents the degeneration of medium spiny neurons in rat corticostriatal brain slices overexpressing the huntingtin exon 1 fragment with a pathogenic repeat,^405^ iron chelator deferoxamine (DFO) benefited striatum pathology and motor phenotype in R6/2 HD mouse,^406^ and deuterium-reinforced linoleic acid, all lowered lipid peroxidation and alleviated cognitive decline in a mouse model of HD (Q140 knock in).^407^

Like AD and PD, HD is also characterised by perturbed mitochondria. Energy metabolism defects were initially implicated due to presentations of weight loss at early stages in patients with HD.^408,409^ Using localized proton nuclear magnetic resonance spectroscopy, bioenergetic dysregulation was further evidenced by elevations of lactate in the occipital cortex of symptomatic HD patients that correlated with illness duration.^410–412^ Increased lactate may occur due to decreased electron transport chain activity and/or elevated glycolysis. FDG-PET studies have revealed hypometabolism in areas impacted early in disease progression (i.e., caudate, putamen, and cerebral cortex) in HD subjects with symptomatic disease,^413,414^ and in non-symptomatic known HD gene carriers.^414–418^ A reduction of glucose uptake and flux through glycolysis and may possibly reduce PPP flux consequently limiting NADPH production and GSH recycling needed for antioxidant protection.^419^ In contrast, hypermetabolism was observed in the cerebellum and some thalamic nuclei, areas effected later in disease progression.^417^ It has been hypothesised that the increase in glucose metabolism is an attempt to prevent mutant huntingtin toxicity,^420^ potentially via increased metabolic flux to increase NADPH production to fuel anti-oxidant defence. Mitochondrial abnormalities were first observed in post mortem cortical tissue from HD patients in 1978^421^ and have been confirmed in multiple HD mouse models.^422^ Prolonged energy impairment due to Complex II inhibition via 3-nitropropionic acid administration has also been used as model for HD.^421,423–425^

### Multiple sclerosis

Multiple sclerosis (MS) is an autoimmune condition characterised by degeneration of the myelin sheath surrounding axons impacting the brain and spinal cord. Degeneration in the deep grey matter has been associated with clinical disabilities including visual abnormalities, weakness, spasticity, urinary dysfunction, and cognitive symptoms. Iron elevation has been observed in deep grey matter from both MS patient autopsies which showed global neurodegeneration associated with an accumulation of oxidised phospholipids and DNA in neurons, oligodendrocytes and axons^426^ and in living MS patients via MRI.^427^ Iron is a necessary factor for several processes that enable myelination by oligodendrites (i.e., ATP, cholesterol, lipid synthesis and myelin basic protein function).^428^ As a result, oligodendrocytes are the brain cells with the highest iron levels.^429^ However in MS, iron accumulation in microglia and other macrophages suggests a pathological role of iron, implicating ferroptosis vulnerability.^427^ Indeed, Luoqian et al. found that ACSL4 was altered in a genomic database of MS patients, and that a ferroptosis signature of accrued lipid ROS and mitochondrial shrinkage were observed in the experimental autoimmune encephalitis mouse model, one of the most commonly used models for MS.^430^ Both liproxstatin-1 and ACSL4 repression (AAV8-*Acsl4*-KD) improved behavioural phenotypes, reduced inflammation and prevented neuronal cell death. Ferroptosis was proposed to cause neurodegeneration through hyperactivity of T-cells, since RSL3-treated neurons injected into naive recipients significantly exacerbated experimental autoimmune encephalitis pathogenesis.^430^ This study highlights a role for ferroptosis in driving immune-mediated neurodegeneration in MS.^430,431^

Mitochondrial abnormalities including morphological changes (swelling), fragmentation and impaired trafficking are also observed in the autoimmune encephalitis mouse model.^432,433^ In vivo imaging of the mouse spinal cord revealed that depolarisation of both the axonal mitochondria and the axons themselves correlated with neurological function and that mitochondrial abnormalities were most severe in regions of perivascular inflammatory cells.^433^ Mitochondrial dysfunction, including decreased activities of ETC complexes I, III^434^ and IV,^435,436^ has also been widely reported in MS patient neurons. It has been suggested that a bioenergetic insufficiency impacts ion homeostasis, inducing Ca^2+^ mediated axonal degeneration.^434^ Remyelination exerts an enormous energy demand, with estimations suggesting that 1 gram of myelin demands 3.3 × 10^23^ ATP molecules.^437^ Thus, oligodendrocytes have a high mitochondrial oxidative phosphorylation (OXPHOS) rate before and during myelination.^438^ Consequently, bioenergetic insufficiency can promote neurodegeneration in MS.

## Common vulnerabilities and predisposing risk factors for ferroptosis in neurodegeneration

Energy stress, mitochondrial abnormalities and excessive oxidative stress parallels ferroptosis signatures in multiple forms of neurodegeneration with diverse genetic origins (AD, PD, HD and MS).^439^ Ultimately, imported glucose must either flux down through glycolysis and oxidative phosphorylation to produce ATP, or be shunted via the PPP to produce NADPH for antioxidant recycling and/or macromolecule synthesis. This creates a metabolic intersection to either create energy (ATP) or fuel ferroptosis defence (NADPH) from imported glucose. A recent study showed that neurons actively preserve low glycolytic flux via proteolytic destabilization of 6-phosphofructo-2-kinase–fructose-2,6-bisphosphatase-3 (PFKFB3; a key glycolysis promoting enzyme), to increase the PPP and therefore boost antioxidant defence to prevent oxidative stress-induced mitochondrial impairment.^440^ Overexpression of *Pfkfb3* in genetically engineered mouse neurons resulted in an accumulation of anomalous mitochondria, complex I disassembly, bioenergetic deficiency, mitochondrial redox stress and decreased GSH, which translated to accelerated cognitive decline. Importantly, behavioural abnormalities were ameliorated with neuron-specific genetic ablation of mitochondrial redox stress (via expression of mitochondrial matrix-tagged catalase) or brain NAD^+^ restoration (via supplementation of NAD^+^ precursor, nicotinamide mononucleotide).^440^ Ferroptosis was not investigated in this mouse model, however, we hypothesise that the decreased GSH and excessive oxidative stress would promote ferroptosis.

Consequently, we propose that energy stress creates an inherent vulnerability for ferroptosis in neurodegenerative diseases. Several consequences of energy stress may render neurons more susceptible to ferroptosis, including decreased glucose flux through the PPP to produce NADPH, reduced ATP required to synthesise key anti-ferroptosis defence substrate GSH, and/or excessive oxidative stress production via compromised mitochondria. Mitochondrial reactive oxygen species can also mediate PFKFB3 stabilisation,^441^ thus mitochondrial impairment may exacerbate both energy stress and impair PPP flux, compounding to increase vulnerability to ferroptosis.

## Interaction of different regulated cell death pathways in neurodegeneration

Despite ferroptosis being classified as a distinct programmed cell death pathway compared to other regulated death mechanisms (i.e., apoptosis and autophagic death), there are several common proteins and cross-signalling pathways. Some common signalling increases the vulnerability of multiple regulated cell death programs; for example, p53 increases ferroptosis through targeting SLC7A11A, spermidine, N1-acetyltransferase 1 or glutaminase 2, but can also increase apoptosis by directly binding to BAX to increase mitochondrial membrane permeabilization, or combine with anti-apoptotic mitochondrial proteins (i.e., Bcl-2 and Bcl-XL) to induce apoptosis.^442–444^ In contrast, the level of a common protein can differentially regulate different cell death programs; for example, increased BAP1 is associated with increased ferroptosis, but decreased BAP1 can cause apoptosis.^445–447^ Erastin, a common ferroptosis inducer, is also capable of binding VDAC which can influence vulnerability to apoptosis at high doses.^448–452^ While in vitro, a tightly controlled environment with cell-death specific inducers can create the illusion of clean single cell death mechanisms, human diseases are likely to comprise of multiple regulated cell death pathways due to the complexity of interacting signalling pathways. Understanding the interaction and interrelation of regulated cell death pathways will be important in understanding the pathogenesis of neurodegeneration.

## Therapeutic strategies and clinical research strategies

Most therapeutic strategies that inadvertently or intentionally target ferroptosis can be classified into three broad categories 1. Radical trapping agents (RTAs) 2. Iron modulation and 3. Glutathione-dependent redox support. By virtue of reducing the fuel or increasing the defence of ferroptosis, these therapeutic strategies also reduce the cellular demand on NADPH generation. Similarly, therapeutic strategies that target NADPH synthesis (i.e. NAD precursors including NAM, NMN and NR^376,453–457^) may also boost cellular defence against ferroptosis. While increasing ferroptosis sensitivity has been exploited in the oncogenic context,^458–460^ this review will focus on pharmacological approaches that increase ferroptosis defence.

### RTAs

RTAs are recognised as a pivotal approach to inhibit phospholipid peroxidation. RTAs can be further classified as endogenous (vitamin E isoforms,^461^ reduced coenzyme Q10,^462^ BH4^463^ and Vitamin K isoforms^203^; discussed previously) or synthetic (Ferrostatin 1,^1^ liproxstatin-1,^9^ liproxstatin-2,^464^ CuATSM,^465^ UAMC-3203,^466^ SRS11-92, SRS9-11, SRS16-86, UAMC-2418, CFI-4061, CFI-4082,^405,467^ Phenothiazine,^468^ 2-{1-[4-(4-methylpiperazin-1-yl)phenyl]ethyl}-10H-phenothiazine,4-{4-[1-(10H-phenothiazin-2-yl)vinyl]phenyl}morpholine,^469,470^ 3-CF3-8-tBu-PNX^471^; recently reviewed^472^). Endogenous RTAs were identified as protective in GPX4 deficient mice preceding the coining of ‘ferroptosis’ by Conrad’s group who showed that cell death could be abolished by alpha-tocopherol.^71^ Whereas development of synthetic RTAs to target ferroptosis began with ferrostatin-1, which was identified by Stockwell’s group through high-throughput screening of a small molecule library encompassing varied drug-like soluble compounds in the study first defining ferroptosis.^1^ Ferrostatin-1’s RTA capacity was later attributed to the amine group and necessary N-cyclohexyl moiety that enabled anchoring in lipid membranes.^465^ Ferrostatin-1 was first modified to UAMC-2418 by replacing the labile ester with amide or sulfonamide and adding a benzyl ring to NH_2_.^467^ Modification of ferrostatin-1 to improve stability however solubility became an issue. UAMC-3203 included additional solubility enhancing groups showing improved solubility and efficacy and a lack of toxicity in mice.^466,473,474^ At a similar time to ferrostatin-1 development, liproxstatin-1 was identified from a small molecule screening in TAM-inducible gpx4^−/−^ mouse embryonic fibroblasts.^9^ The RTA capacity to block peroxyl radicals was later attributed to the liproxstatin’s quinaxoline ring.^475^ Conrad and coworkers since created liproxstatin-2, which has improved pharmacokinetic properties and efficacy (chemical structure not disclosed).^464^ Despite efficacy in inhibiting ferroptosis, non-RTA ferroptosis inhibitors are often perceived as more druggable due to their specific protein targets.^472^

### Iron modulation

Since iron can contribute to lipid peroxidation both enzymatically and non-enzymatically,^476^ iron chelation has emerged as a common anti-ferroptotic therapeutic strategy. Iron chelation involves the use of ligands that bind iron before being excreted from the body via stool or urine to reduce tissue iron.^477,478^ Common iron chelators used to prevent ferroptosis include deferoxamine, deferiprone and deferasirox, which have previously been developed and tested for iron overload disorders such as hemochromatosis, beta-thalassemia and sickle cell disease.^421,479,480^ Deferoxamine chelates ferric iron at a 1:1 stoichiometry enabled by a chain of 3 hydroxamic acids terminating in a free amino acid group.^481^ However, the hydrophilic structure of deferoxamine results in poor absorption and rapid metabolism which forces frequent parenteral administration in patients (over 8 to 12 h per day), thus imposing a significant treatment burden.^479,482^ Deferiprone overcame treatment burdens and was the first orally active iron chelating drug, however was associated with gastrointestinal side effects.^483^ Deferasirox on the other hand can coordinate iron to for a 2:1 stoichochemistry, enabled by a triazolyl nitrogen and two phenolic oxygens as donor groups. Deferasirox can also conveniently be taken orally, but has been associated with hepatic, gastrointestinal and renal toxicities.^484^

MRI studies have confirmed the potential of deferiprone to cross the blood brain barrier and remove iron from the brain, leading to the hypothesis that iron chelation may slow down the progression of neurodegenerative diseases associated with elevated iron.^485^ Indeed, in a preliminary study, deferiprone treatment for 12 months (30 mg per kilogram per day) in patients with early-stage PD experienced a decrease in both substantia nigra iron deposits and motor-scale indicators of disease progression.^486^ However, in a follow up multi-centre phase 2 trial in participants with early Parkinson’s disease, deferiprone was associated with worse scores in measures of parkinsonism than those with placebo over a period of 36 weeks.^487^ The contradictory results of iron chelation in PD may be explained by the difference in dopaminergic medications. In the preliminary study, subjects remained on stabilised L-DOPA regimes, however in the phase 2 multicentre trial, subjects were not treated with L-DOPA. Iron is a cofactor for tyrosine hydroxylase, an enzyme required for dopamine serotonin synthesis.^488^ Thus, iron chelation may have limited dopamine synthesis, confounding any potential benefit of iron chelation in preventing ferroptosis. However, iron also plays a key role in energy metabolism; in the form of iron sulfur clusters, iron enables electron transport to drive oxidative phosphorylation and energy production. As previously discussed, decreased energy production is a hallmark of several neurodegenerative diseases thus caution is advised in using iron-chelation. As a double edge sword, iron chelation may prevent labile iron catalysing ferroptosis, but may also disrupt energy production. Ramifications of reducing iron-overload via iron chelation in a highly metabolically active tissue was evidenced in a limb ischemic reperfusion mouse model.^89^ Despite showing hallmarks of ferroptosis (excess iron and lipid peroxidation), mice treated with deferiprone experienced an exacerbated ischemic reperfusion injury to hindlimb muscle. Recently, iron chelation in an Alzheimer’s disease clinical trial similarly exacerbated disease pathology raising questions about the suitability of iron chelation in complex in vivo systems, particularly when energy demand is high, or energy impairment is characteristic of the condition {Ayton, 2024 #1578}.

### Glutathione-dependent redox support

*N*-acetylcysteine (NAC) has been shown in several in vitro studies to inhibit ferroptosis by targeting cysteine and GSH metabolism.^489–493^ NAC has also been clinically shown to improve neurodegeneration-related symptoms in PD patients.^494^ However poor bioavailability and blood-brain barrier permeation have been implicated in the inconsistency of several clinical trial results. To overcome these limitations of NAC, an amide derivative N-Acetylcysteine amide (NACA) has been created to advance the lipophilicity, membrane permeability, and antioxidant property to increase stability and blood brain barrier penetration.^495,496^ Selenium supplementation is another approach to increase glutathione-mediated ferroptosis defence.^163,336,497,498^ There is currently a large phase II randomised, double-blind, placebo-controlled trial of sodium selenate as a disease-modifying therapy for behavioural variant frontotemporal dementia.^499^ This clinical trial is based on a proposed mechanism that sodium selenate acts as a specific agonist for PP2A, one of the implicated phosphatases in regulating tau protein phosphorylation.^500^ As a result, sodium selenate-treated transgenic TAU441 mice had significantly reduced phospho- and total tau levels (hippocampus and amygdala), and demonstrated improvements in spatial learning and memory.^500^ However, this does not rule out a complimentary GPX4 boosting affect.

#### Drug repurposing to discover novel ferroptosis therapeutics

Drug repurposing offers a potentially expedited path to clinical trials at lower costs and reduced safety risks.^501^ Recently, 1176 FDA approved drugs were screened using erastin for ferroptosis inhibitors as neuroprotective agents.^502^ 89 drugs showed anti-ferroptotic activity and the top 26 drugs with EC_50_ values below 10 μM were further investigated to categorise mechanistic activity. Most of the drugs scavenged free radicals (*n* = 25) while a subset chelated Fe^2+^(*n* = 6) and inhibited 15-LOX (*n* = 6). 11 of the top 26 drugs (lumateperone tosylate, eltrombopag olamine, osimertinib, isoetharine mesylate salt, tizoxanide, indacaterol, clofazimine, indacaterol, valrubicin and fenoldopam mesylate) were newly identified and hold promise for further characterisation in bona fide ferroptosis disease models. Four of the top 26 drugs were antipsychotics (lumateperone, promethazine, thioridazine and olanzapine), with EC50s < 4 µM. Lumateparone was the most potent of the 26. This is curious because recent work has identified an elevation of iron in the brain in schizophrenia^503,504^ and bipolar disorder.^505^

Ferroptosis has been implicated in a wide range of diseases as previously discussed and the list will continue to grow with ongoing clinical trials investigating biomarkers of ferroptosis in other contexts (e.g., air pollution exposure NCT05753332/ NCT05758129, Myelodysplastic syndromes NCT05924074, Vitiligo NCT06261086, Lead exposure NCT05950386, Lymphedema NCT06237907 and epilepsy NCT05269901). 16 Clinical trials associated with ferroptosis were identified on clinicaltrials.gov using a filter of key search term ‘ferroptosis’ (Table 1).

**Table 1:** Clinical trials associated with ferroptosis

Clinical trial ID	Status	Title	Study objectives	Key Outcomes
NCT05753332	Active, Not recruiting	Association Between Short-term PM2.5 Exposures and Nrf2 Dependent Ferroptosis Pathway	Discover of the effects of air pollution (PM2.5) on the Nrf2- dependent ferroptosis pathway	To be confirmed (TBC)
NCT05758129	Active, Not recruiting	Association Between PM2.5 Exposure and Ferroptosis in Seizures Patients	Discover the possible effects of PM2.5 exposures on the Nrf2- dependent ferroptosis pathway in seizure patients	TBC
NCT05493800	Active, Not recruiting	Evaluate the Safety and Efficacy for Oral Mucositis Prevention of MIT-001 in Auto HSCT (Capella) ClinicalTrials.gov ID NCT05493800	Evaluate the efficacy and safety for the prevention of oral mucositis and PK of MIT-001 (a ferroptosis inhibitor^506^) for lymphoma or multiple myeloma patients receiving conditioning chemotherapy for autologous hematopoietic stem cell transplantation(auto-HSCT).	TBC
NCT05924074	Not yet recruiting	Ferroptosis Study in SF3B1-mutant Myelodysplastic Syndromes (FerMDS) (FerMDS)	Ferroptosis will be analyzed using flow cytometry (labelling of peroxidized lipids with C11-BODIPY) from bone marrow samples from patients with Myelodysplastic syndromes (MDS): clonal diseases of hematopoietic stem cells characterized by dysplastic and inefficient hematopoiesis related to excessive progenitor cell death.	TBC
NCT06491394	Not yet recruiting	Lactoferrin Effect on Kidney and Heart of Rhabdomyolysis Rats	To investigate the possible beneficial effect of Lactoferrin administration on the kidney and the heart of glycerol-induced rhabdomyolysis in rats and its relation to ferroptosis and AMPK/Nrf2/ HO-1 signalling pathway.	TBC
NCT06218524	Not yet recruiting	The Effectiveness of HP and TMZ Synergism on Adult Recurrence GBM	To investigate the potential of haloperidol (an antipsychotic drug) to reverse ferroptosis resistance triggered by Temozolomide in patients who suffer from recurrent glioblastoma.	TBC
NCT04211636	Not yet recruiting	Autoimmunity And Immune Deficiency After Spinal Cord Injury: Association With Rehabilitation Outcomes	To systematically analyze humoral autoantibody responses and their interaction with post-spinal cord injury immune-deficiency and infections as well as their association with the clinical course of rehabilitation and markers of ferroptosis in plasma and CSF.	TBC
NCT06261086	Not yet recruiting	Evaluation of Pyroptosis-related Indicators in the Pathogenesis of Vitiligo: Across-sectional Comparative Study	To investigate necroptosis, pyroptosis, and ferroptosis markers in serum to delineate a role of cell death pathways in the pathogenesis of Vitiligo, an acquired pigmentary disorder on skin and/or mucosae, which is characterized by death of melanocytes.	TBC
NCT05950386	Recruiting	Effects of Lead Exposure on Ferroptosis Pathway	Investigate the effects of chronic lead exposure on mRNA levels of genes associated with iron metabolism (FTH1, FPN1, DMT1) and the Nrf2-dependent ferroptosis pathway (Nrf2, SLC7A11, GPX4) in blood samples from lead acid battery factory workers.	TBC
NCT06237907	Recruiting	Pyroptosis and Ferroptosis in the Pathophysiology of Lymphedema	Compare the differences in the expression of cell death through apoptosis and iron-dependent cell death in the subcutaneous adipose tissue after the reduction of edema symptoms following lymphedema surgery in patients	TBC
NCT06102993	Recruiting	Ferroptosis in Patients With COPD With/Without Risk of Cardiovascular Events. Pathophysiological Implications, Diagnostics and Prognoses. FerrEPOC Study. (FerrEPOC)	To investigate a small group of circulating proteins in blood previously identified using Differentially Expressed Genes (DEGs) related to ferroptosis that overlap with the DEGs of COPD and the DEGs of atherosclerosis to evaluate the relationship between these molecules and clinical variables of COPD and their potential utility in identifying the risk of exacerbations, admissions, and cardiovascular events in COPD.	TBC
NCT06048367	Recruiting	Carbon Nanoparticle-Loaded Iron [CNSI-Fe(II)] in the Treatment of Advanced Solid Tumor (CNSI-Fe(II))	evaluate the safety, tolerability, pharmacokinetics (PK) profile, dose and preliminary efficacy of intratumoral injection of Carbon Nanoparticle-Loaded Iron [CNSI-Fe(II)] (to induce ferroptosis^507^) in patients with advanced solid tumors.	TBC
NCT06151548	Recruiting	Effect of Krill Oil Supplementation on Red Blood Cell Physiology Against Changes in Markers of Iron Metabolism.	To investigate whether supplementation with krill oil may have a beneficial effect on athletes by limiting lipid peroxidation and inhibiting ferroptosis which in consequence may lead to red blood cell membrane protection.	TBC
NCT05410665	Unknown	The Roles of IL-9/E-cadherin and Ferroptosis in Intestinal Mucosal Barrier Injury in Sepsis	Evaluate the roles of IL-9/E-cadherin and ferroptosis in the intestinal mucosal barrier injury of sepsis.	TBC
NCT05269901	Completed	Association Between Ferroptosis and Epilepsy	Obtain peripheral blood from 20 newly epileptic diagnosed untreated school-aged children (6 –12 years) and 20 age-matched healthy controls to investigate three glutathione peroxidase 4 (GPX4) dependent ferroptosis pathway biomarkers: Nrf2, SLC7A11, and GPX4.	mRNA expression levels of Nrf2, SLC7A11, and GPX4 were significantly reduced in peripheral blood from patients with newly diagnosed and untreated seizures suggesting that the Nrf2-mediated ferroptosis pathway might be associated with the occurrence of seizures in a clinical setting.^508^
NCT04378075	Completed	A Study to Evaluate Efficacy and Safety of Vatiquinone for Treating Mitochondrial Disease in Participants With Refractory Epilepsy (MIT-E)	To investigate the efficacy and Safety Study of Vatiquinone (ferroptosis inhibitor^509^) for the Treatment of Mitochondrial Disease Subjects With Refractory Epilepsy	Not yet posted to Clintrials.gov; press release by PTC Therapeutics June 2023 reported failure to achieve primary endpoint but significantly reduced seizure incidence in the subset of patients with Leigh syndrome.^510^

#### Conclusion, future directions and open questions

Since ferroptosis was coined in 2012, the field has grown rapid momentum. Over the last decade, the development of a range of ferroptosis induces and rescuers, (oxidised) lipidomic techniques and CRISPR knock out screens has enabled the field to create a sound understanding of the biochemical pathways underpinning ferroptosis initiation and defence. This review focuses on key ferroptotic defence enzymes and metabolites; TR, GR, FSP1, DHFR, NQO1 and retinal reductases, and identified a common thread: a dependence on a reducing equivalent for redox recycling. NADPH is the major reducing agent of the cell, and its abundance is dependent on the complex interaction between metabolic pathways that control fundamental life processes including energy production, molecule synthesis and antioxidant defence. This leads us to pose whether NADPH or downstream protein/metabolites form the foundation of ferroptosis defence in disease?

Parallel emerging evidence in ischemic reperfusion and neurodegenerative diseases support the presence of both ferroptosis and metabolic dysregulation centred around NADPH and metabolite resources required to synthesise and recycle NADPH. Ferroptosis research has been recurrently linked to neurodegeneration. Still, while a wealth of literature suggests a ferroptotic component in neuronal cell death, we lack a granular understanding of how ferroptosis can be activated in the presence of its main guardian – glutathione peroxidase 4 (GPX4). Ferroptosis is classically induced by impaired cystine/cysteine transport (leading to GSH loss) or direct GPX4 inhibition. Yet, it is unclear if this occurs in any common chronic disease. This disconnect between how ferroptosis is modelled within the laboratory, and the pathophysiology of chronic disease has been a barrier to translation of ferroptosis concepts and therapeutics into the realm of clinical research. Given the foundational role of NAPDH in ferroptosis defence, this leads us to question whether ferroptosis may be unleashed by metabolic dysregulation leading to altered carbon flux and failure to supply enough NADPH to regenerate GSH and other ferroptosis suppressors.

The translation of ferroptosis theories into therapies is currently limited by a lack of understanding of physiological triggers in the context of human disease. While in-vitro and genetic animal models have demonstrated molecular pathways downstream of ferroptosis induction, the next frontier for the ferroptosis field will involve understanding the causes of ferroptosis in human diseases. It is likely that induction of ferroptosis in disease will occur through the erosion of ferroptosis defences that are ineffectually restored by disrupted or fatigued metabolic pathways. By understanding the metabolic pathway disruption and other pathophysiological determinants that dismantle ferroptosis defence, this may expose druggable targets to restore metabolic flux and recover ferroptosis defences in disease contexts.
